# Phosphorylated CpxR Restricts Production of the RovA Global Regulator in *Yersinia pseudotuberculosis*

**DOI:** 10.1371/journal.pone.0023314

**Published:** 2011-08-18

**Authors:** Junfa Liu, Ikenna R. Obi, Edvin J. Thanikkal, Thomas Kieselbach, Matthew S. Francis

**Affiliations:** 1 Department of Molecular Biology, Umeå University, Umeå, Sweden; 2 Umeå Centre for Microbial Research, Umeå University, Umeå, Sweden; 3 Department of Chemistry, Umeå University, Umeå, Sweden; 4 College of Veterinary Medicine, Huazhong Agricultural University, Wuhan, People's Republic of China; Universidad Nacional, Heredia, Costa Rica

## Abstract

**Background:**

RovA is a global transcriptional regulator of gene expression in pathogenic *Yersinia*. RovA levels are kept in check by a sophisticated layering of distinct transcriptional and post-transcriptional regulatory mechanisms. In the enteropathogen *Y. pseudotuberculosis*, we have previously reported that the extracytoplasmic stress sensing CpxA-CpxR two-component regulatory system modulates *rovA* expression.

**Methodology/Principal Findings:**

In this study, we characterized CpxR phosphorylation (CpxR∼P) *in vitro*, and determined that phosphorylation was necessary for CpxR to efficiently bind to the PCR-amplified upstream regulatory region of *rovA*. The precise CpxR∼P binding site was mapped by a nuclease protection assay and directed mutagenesis confirmed that *in vivo* binding to the *rovA* promoter inhibits transcription. Reduced RovA production was most pronounced following CpxR∼P accumulation in the *Yersinia* cytoplasm during chronic Cpx pathway activation and by the indiscriminate phosphodonor action of acetyl phosphate.

**Conclusions/Significance:**

Cpx pathway activation restricts levels of the RovA global regulator. The regulatory influence of CpxR∼P must therefore extend well beyond periplasmic quality control in the *Yersinia* envelope, to include genes involved in environmental survival and pathogenicity.

## Introduction

Extracytoplasmic stress (ECS) has a deleterious effect on bacterial fitness, since it impacts on cell envelope integrity, protein folding and function. Bacteria have consequently evolved multiple signaling pathways to counter these situations [1]. One of these is a two-component regulatory system (TCRS) composed of CpxA and CpxR (for conjugative plasmid expression). In response to diverse stresses (such as high pH, detergents, EDTA, altered membrane lipid composition and bacterial adhesion to surfaces) that ultimately leads to protein misfolding in the periplasm, the Cpx pathway induces synthesis of periplasmic protein folding and degradation factors that aid in the (re)folding of extracytoplasmic proteins and protein complexes [2], [3], [4].

Spanning the inner membrane, CpxA is a histidine kinase possessing three distinct activities in *E. coli* – autokinase, CpxR kinase and CpxR phosphatase activity [5]. In the absence of inducing signal, CpxA activity is subdued by the binding in the periplasm of the accessory factor CpxP [6]. In recognition of ECS signals however, CpxP is titrated away from CpxA and is targeted for degradation by the DegP serine protease [7], [8]. This enables CpxA to become auto-phosphorylated presumably at the conserved histidine residue at position 249 [5]. This permits the phosphate to be relayed to the CpxR response regulator that, based on homology to other response regulators, would occur on the aspartate residue at position 51 [9]. At least in *E. coli*, CpxR∼P is then capable of up- or down-regulating the transcription of many genes that function to alleviate the effects of ECS [2], [3], [4]. Once this has occurred, status quo is restored by the phosphatase activity of CpxA, which de-phosphorylates CpxR [5]. Thus, a consequence of generating CpxA phosphatase deficient *E. coli*, as would result from a full length *cpxA* deletion mutant or so-called gain-of-function mutants (designated cpxA*), would be to accumulate CpxR∼P [5], [10], [11], [12], [13], [14]. In these situations, acetyl phosphate (acetyl∼P), a small molecular weight phosphodonor, can also elevate CpxR∼P levels [5], [15], [16], [17]. This CpxA-independent phosphorylation of CpxR by acetyl∼P is potentially a global signal reflecting the status of bacterial growth and central metabolism [18].

Homologues to components of the Cpx pathway exist in a number of clinically important bacterial pathogens. We have recently studied the function of the Cpx TCRS in the enteropathogen *Yersinia pseudotuberculosis* [19], [20], a bacterium causing self-limiting gastroenteritis in infected individuals [21]. Intriguingly, loss of CpxA in this bacterium caused a down-regulation of several determinants known to be important in the pathogenesis of disease in mouse infection models; most notably, the plasmid-encoded Ysc-Yop type III secretion system (T3SS), an integral outer membrane adhesin, termed invasin, and RovA, a member of the MarR/SlyA family of transcriptional regulators [19], [20]. These studies provided the first indications that CpxR∼P could act as a negative transcriptional regulator of *Y. pseudotuberculosis* virulence factors. The fact that we could demonstrate *in vitro* CpxR∼P binding to *inv* and *rovA* promoters supported this view, although typical consensus CpxR∼P binding sites in these regions were never located [20]. Moreover, it was assumed from this earlier work that *Y. pseudotuberculosis* lacking CpxA phosphatase activity must accumulate CpxR∼P, serving as the fundamental basis for transcriptional repression of virulence gene expression in this mutant background [19], [20]. However, this critical notion is unproven.

RovA was first identified in *Yersinia* because it was required for transcription of the *inv* gene encoding for invasin [22], [23]. Whole-genome analyses has since indicated a global regulatory role for RovA in pathogenic *Yersinia* [24], [25], [26]. Additional virulence strategies among the RovA regulon include the prominent pH 6 antigen chaperone-usher system, the Ysc-Yop T3SS, and other uncharacterized potential secretion systems. To regulate these virulence determinants, RovA levels are therefore tightly controlled by multiple pathways in accordance with the prevailing environmental growth conditions. Dynamic regulatory mechanisms are obviously paramount for RovA to impart significant global control on virulence gene transcription in *Yersinia*. Thus, this study therefore seeks to substantiate the contribution of the Cpx TCRS to control of RovA-dependent virulence gene regulation in *Yersinia*.

## Materials and Methods

### Bacterial strains, plasmids and growth conditions

Bacterial strains and plasmids can be viewed in Table S1. We consistently used *Y. pseudotuberculosis* YPIII/pIB102 (serotype III) as the parental strain. pIB102 is a virulence plasmid encoding for the Ysc-Yop T3SS and is a variant of cryptic pIB1. It differs only by a kanamycin resistance cartridge inserted into the *yadA* gene, which does not reduce the pathogenicity of YPIII/pIB102 in mouse models [27]. The strain also harbors an internal duplication within *phoP* located on the chromosome. This is predicted to generate an inactive truncated variant of the PhoP response regulator that impairs bacteria growth and survival inside macrophages [28]. Bacteria were normally cultivated in Luria-Bertani (LB) agar or broth at either 26°C (*Y. pseudotuberculosis*) or 37°C (*E. coli*) with aeration. When examining transcription and translation from *rovA* and *inv*, bacteria were grown at both 26°C and 37°C in LB broth to late stationary phase. These conditions were selected on the basis that reports from other laboratories have clearly established the peak *rovA* and *inv* expression occurs in bacteria grown at low temperature to stationary phase [22], [24]. Where required, antibiotics were added at the final concentrations of carbenicillin (Cb; 100 µg per ml), kanamycin (Km; 50 µg per ml), Trimethoprim (Tp; 10 µg per ml) and chloramphenicol (Cm; 25 µg per ml).

### Mutant construction

To construct individual in-frame deletions, site-directed amino acid substitutions and nucleotide ‘shuffle’ mutations, we applied the standard overlap PCR technique using the relevant primer combinations listed in Table S2. To facilitate the sequencing process (performed by MWG Biotech AG, Ebersberg, Germany), all amplified fragments were initially cloned into pCR®4-TOPO TA (Invitrogen AB, Stockholm, Sweden). Confirmed fragments were then lifted into the mutagenesis vector, pDM4. These mutagenesis constructs were then conjugated by *E. coli* S17-1λ*pir* into *Y. pseudotuberculosis*. Mutated alleles were initially introduced into the recipient genome by a single cross-over event. A second single cross-over event followed to secure the complete allelic exchange, which was initially screened for on the basis of *sacB*-dependent sucrose sensitivity [19], [29]. The presence of the mutated allele in the *Y. pseudotuberculosis* genome was then confirmed by diagnostic PCR and sequence analysis of the amplified regions flanking the mutation.

### Western blotting

Protein derived from lysates of the bacterial pellet was fractionated by 12% (for invasin) or 15% (for RovA) SDS-PAGE. Protein was then transferred to Schleicher and Schuell Protran® nitrocellulose (GE Healthcare) using a Hoefer semi-dry transfer assembly. Proteins of interest were bound with specific rabbit polyclonal antibodies that were a gift from Petra Dersch (anti-RovA), Hans Wolf-Watz (anti-invasin), Thomas Silhavy (anti-MBP-CpxR) or Shu-ichi Nakayama (anti-CpxR). These were then detected with an anti-rabbit monoclonal antibody conjugated with horse radish peroxidase (GE Healthcare) and a homemade chemiluminescent solution.

### *nlpE* cloning and expression

The primer combination for PCR amplification of *Y. pseudotuberculosis nlpE* is listed in Table S2. The DNA fragment was cloned into pBAD18 [30] using *EcoRI/XbaI* restriction to generate pJF027. This placed *nlpE* expression under arabinose control. The *nlpE* allele from *E. coli* cloned under arabinose control in pBAD18 (termed pND18) was a gift from Thomas Silhavy. These two constructs along with the vector control were electroporated into *Yersinia*. To induce *nlpE* expression, overnight bacterial cultures were used to seed fresh growth medium supplemented with 0.2% (w/v) L-arabinose and appropriate antibiotic selection.

### Cloning, expression and purification of CpxR variants

Alleles encoding CpxR wild type and the CpxR_D51A_ and CpxR_M199A_ variants were amplified with gene specific primers (Table S2) using template DNA derived from parental *Y. pseudotuberculosis* or the respective mutants and cloned with *NdeI* and *XhoI* in front of the IPTG inducible promoter of pET22b(+). These expression plasmids were maintained in *E. coli* BL21(DE3) plysS allowing for the IPTG-inducible expression of individual CpxR variants fused to a C-terminal His_(6)_ tag. Recombinant protein was purified as previously described [20].

### Electrophoretic mobility shift assay (EMSA)

^32^P end-labeled forward primers listed in Table S2 were used for the PCR amplification of the promoter regions of *cpxR/P*, *rovA*, *ppiA*, *ail*, *ail*-like, *inv*, *psaA* and *psaE*. The amplified DNA fragments were purified by agarose gel electrophoresis. Purified CpxR was mixed with the DNA fragments (∼2 to 5 nM) in a 20 µL reaction volume containing 20 mM Tris-HCl pH 7.0, 30 mM Acetyl phosphate, 125 mM KCl, 10 mM MgCl_2_, 1 mM EDTA, 1 mM dithiothreitol, 0.25 mg per ml BSA and 5 µg per ml sonicated salmon sperm DNA. BSA corresponding to the highest CpxR concentration (1.5 µM) was used as a negative control, and about 7 fold excess of unlabeled PCR amplified DNA was used in a competition reaction. After incubation at 30°C for 1 h and addition of the DNA dye solution (40% glycerol, 0.05% bromophenol blue, 0.05% xylene cyanol), the mixture was loaded directly onto a pre-run 5% polyacrylamide gel. Gel electrophoresis was performed in 1× TBE at 100 V for 2 h at 4°C. After gel drying, signals were detected by autoradiography with a Storm 860 PhosphorImager (Molecular Dynamics).

### DNase 1 footprinting assay

The primers pE-rovAfor3, pE-ppiAfor, pcpxRb and pcpxRfor were radioactively labeled with ^32^P using γ^32^P-ATP (Perkin Elmer) and T4 polynucleotide kinase (Fermentas). The labeled pE-rovAfor3, pE-ppiAfor and pcpxRb were paired with unlabeled pE-rovArev3, pE-ppiArev and pcpxPb respectively and used to PCR amplify promoter regions of *rovA* (314 bp), *ppiA* (276 bp) and the divergent *cpxR-cpxP* (245 bp). For a control, the labeled pcpxRfor was paired with unlabeled pcpxRrev for the PCR amplification of an internal region of *cpxR* (389 bp). Subsequently, 1.5 nM of the amplified DNA fragments and 0, 50, 100, 200 and 400 nM acetyl∼P phosphorylated CpxR_wt_::His_6_ were mixed in a 40 µL reaction containing 25 mM Hepes (pH 8), 100 mM potassium glutamate, 0.5 mg/mL BSA. After incubation of the reaction mixture at room temperature for 10 minutes, 4 µl DNase 1 (0.0005 mg/mL; Sigma) was added and the reaction was left for 80 seconds at 37°C. The DNase 1 digestion was stopped by addition of phenol/chloroform and 200 µL DNase 1 STOP (0.4 M sodium acetate (pH 5), 2.5 mM EDTA, 10 ug/mL herring sperm DNA), and then the DNA was precipitated using ethanol. Samples were analyzed on a 7% denaturing polyacrylamide gel and visualized with a Storm 860 PhosphorImager (Molecular Dynamics).

### Visualization of phosphorylated CpxR (CpxR∼P)

Purified CpxR variants labeled with acetyl∼P were mixed with 2× loading buffer (0.02% (w/v) Bromophenol blue, 20% (v/v) Glycerol). For analysis, samples were either fractionated on a SDS-12%-PAGE or a Manganese(II)-Phos-tag™ (33 µM) 12.5% acrylamide gel. Purified CpxR∼P was detected by the Pro-Q® Diamond phosphoprotein gel staining method as described by the manufacturer (Invitrogen). To confirm equal loading, subsequent detection of total protein present on the same gel was revealed by SYPRO® Ruby staining (Invitrogen). In both cases, fluorescent output was recorded using a Fluor-S™ MultiImager (BioRad) and band intensity quantified with Quantity One® quantitation software version 4.2.3 (BioRad). The presence of recombinant purified CpxR∼P or that which is produced endogenously and present in bacterial lysates was also assessed by coupling Manganese(II)-Phos-tag™ acrylamide gel fractionation to immunoblotting with monospecific polyclonal anti-CpxR antiserum. The Manganese(II)-Phos-tag™ approach is an affinity based system for the recognition and separation of anionic substrates, such as phosphorylated proteins, during polyacrylamide electrophoresis [31]. For example, characteristic separation patterns for phosphoprotein isoforms can be generated according to the number and or site of the phosphate group. As a result, it later proved to be routinely applicable to *in vitro* and *in vivo* visualization of TCRS response regulator aspartate phosphorylation [32]. Samples for this analysis were generated from pelleted bacteria vigorously resuspended in 1.2 M formic acid preceding a very brief incubation at room temperature. Prior to fractionation, lysates were solubilized by addition of 4× loading buffer (250 mM Tris-HCl pH 6.8, 8% SDS, 40% glycerol, 4% BME, and 0.08% Bromophenol Blue,) and neutralized by the addition of minuscule amounts of 5 M NaOH.

### Biophysical analysis of intact and digested CpxR∼P

The reduction of CpxR∼P using sodium borohydride was performed as described previously [33], [34]. Before the reaction, CpxR∼P was desalted using C_18_ micro columns and dried by vacuum centrifugation [35], [36]. Controls were performed using unphosphorylated CpxR under identical conditions.

Intact mass determination of CpxR and CpxR∼P utilized purified recombinant CpxR, and this CpxR phosphorylated with acetyl∼P. Duplicate samples of both phosphorylated and non-phosphorylated CpxR were then desalted on homemade C_18_ columns [35], [36]. For analysis by ESI-MS, the proteins were dissolved in 50% (v/v) acetonitrile containing 0.5% (v/v) formic acid. ESI-MS spectra of intact CpxR and CpxR∼P were acquired by ESI-MS in the positive ion mode using a Q-Tof ultima mass spectrometer (Waters, Manchester, UK). Spectra were acquired off-line using nano spray capillaries (Q-tof) from Proxeon (Odense, Denmark) and deconvoluted using the MassLynx 4.0 software.

Peptides of CpxR and CpxR-P were prepared using trypsin and pepsin. Protein was desalted using C_18_ micro columns and dried by vacuum centrifugation [35], [36]. In-solution digestion using trypsin was performed for 40 min at 37°C in 10 µl of fresh 50% ammonium bicarbonate containing 10 ng/µl of sequencing grade trypsin (Promega Biotech AB, Nacka, Sweden). In-solution digestion using pepsin (pepsin from porcine gastric mucosa, Sigma Life Sciences) was performed for 40 min at 37°C in 20 µl of 5% (v/v) formic acid containing 20 ng/µl of pepsin. As an alternative protocol, in-solution digestion using pepsin was performed for 10 min at 37°C in 20 µl of 5% (v/v) formic acid containing 100 ng/µl of pepsin. The generated peptides were then purified using C18 micro-columns [35], [36] loaded with Poros R3 C_18_ material (Applied Biosystems, Stockholm, Sweden) and dried by vacuum centrifugation. In addition, enrichment and purification of phosphorylated peptides was performed by affinity chromatography on titanium dioxides as described [37], [38], [39].

MALDI-MS of peptides were acquired using a Voyager DE-STR mass spectrometer (AB SIEX. Stockholm, Sweden) in the reflector mode and 2,5 dihydroxybenzoic acid (DHB) solution containing 0.5% (v/v) phosphoric acid as a matrix (2,5 dihydroxybenzoic acid solution G2039A) from Agilent Technologies, Dalco Chromtech AB, Sollentuna, Sweden. LC-MS/MS combined with ESI-TRAP and ETD-TRAP was performed using a HCT ultra ETD II mass spectrometer from Bruker linked to Easy nano LC from Proxeon. Spectra were acquired using the enhanced scanning mode covering a mass range from m/z 200 to m/z 1300.

### Analysis of gene transcription by Reverse Transcription (RT)-PCR

Protocols detailing the isolation of total RNA, the reverse transcription of mRNA into cDNA and its use as template for subsequent PCR amplification with the gene specific primers listed in Table S2 are described in detail elsewhere [19], [20].

## Results

### Phosphorylation of the CpxR residue Asp_51_ by acetyl∼P

**Figure 1:**
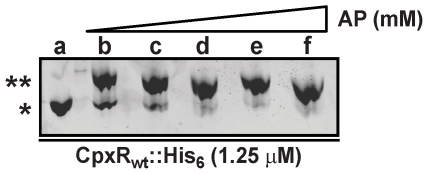
*In vitro* phosphorylation of CpxR by acetyl∼P. Wild type CpxR (CpxR_wt_::His_6_) was purified by metal affinity chromatography. Purified protein (1.25 µM) was incubated in sequentially increasing concentrations of acetyl∼P (AP). Samples were fractionated on a Manganese(II)-Phos-tag™ 12.5% acrylamide gel and then visualized by the SYPRO® Ruby staining method. Lanes: a, no addition of AP; b, 6.25 mM AP; c, 12.5 mM AP; d, 25 mM AP; e, 50 mM AP; f, 100 mM AP. Phosphorylated CpxR is indicated by a double asterisk and unphosphorylated CpxR by a single asterisk.

Phosphorylation of response regulators is thought to stimulate structural changes leading to formation of functional dimers – a prerequisite for efficient binding to target DNA. Thus, to determine if CpxR∼P truly mediates repression of *Yersinia* virulence, we first wanted to characterize CpxR phosphorylation *in vitro* using a high energy phosphate donor, acetyl∼P. We initially determined the minimal concentration of acetyl∼P needed to sufficiently *in vitro* phosphorylate CpxR. Purified CpxR_wt_::His_6_ was incubated with increasing concentrations of acetyl∼P. Aliquots were then fractionated on Manganese(II)-Phos-tag™ 12.5% acrylamide gels to monitor the extent of CpxR∼P. As little as 6.25 mM acetyl∼P was sufficient to phosphorylate the majority of CpxR (Figure 1). Moreover, the maximal achievable amount of CpxR∼P was reached in the presence of 25 mM acetyl∼P (Figure 1). Hence, *in vitro* phosphorylation of CpxR by acetyl∼P is a robust method for generating a high proportion of CpxR∼P molecules.

**Figure 2:**
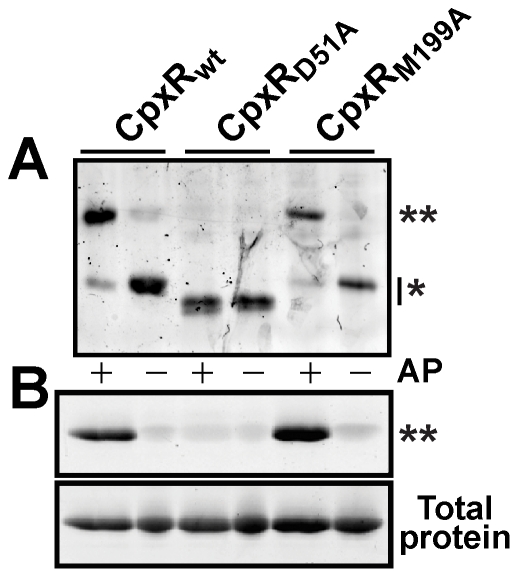
Phosphorylation of the CpxR residue Asp_51_. Wild type (CpxR_wt_::His_6_) and defined variants with the amino acid substitutions Asp_51_Ala (CpxR_D51A_::His_6_) and Met_199_Ala (CpxR_M199A_::His_6_) were purified by metal affinity chromatography. Purified proteins (1.25 µM) were incubated in the presence of 50 mM acetyl∼P (AP) (+). In A, samples were fractionated by a Manganese(II)-Phos-tag™ 12.5% acrylamide gel and then stained by the SYPRO® Ruby staining method. In B, samples were separated in a conventional SDS 12% polyacrylamide gel. Phosphorylated protein (upper panel) was specifically detected by the Pro-Q® Diamond phosphoprotein gel stain technique, while total protein (lower panel) was visualized by the SYPRO® Ruby staining method. Phosphorylated CpxR is indicated by a double asterisk and unphosphorylated CpxR by a single asterisk. The reason for the enhanced mobility of CpxR_D51A_::His_6_ when fractionated by Manganese(II)-Phos-tag™ acrylamide (Panel A) is uncertain.

On the basis of homology to the OmpR/PhoB response regulator family, the conserved aspartate at position 51 (Asp_51_) is the predicted phosphorylation site of CpxR [9]. We therefore established a *cpxR* mutation in which Asp_51_ was replaced with alanine. As a control, another mutant was constructed in which methionine at position 199 (Met_199_) was replaced with alanine. Based on the helix-turn-helix DNA-binding motif prediction algorithm of Dodd and Egan [40], this substitution is predicted to disrupt a helix-turn-helix motif needed for DNA binding – a characteristic of the OmpR/PhoB family [41] (data not shown). C-terminal His_6_-tagged fusions of CpxR_wt_ and the two CpxR_D51A_ and CpxR_M199A_ variants were then purified by affinity chromatography from *E. coli*. Acetyl∼P was used to investigate which of these purified proteins could still be phosphorylated. The Manganese(II)-Phos-tag™ acrylamide system was used to visualize phosphorylated protein. Only CpxR_wt_::His_6_ and CpxR_M199A_::His_6_ were deemed to be phosphorylated *in vitro* by acetyl∼P (Figure 2B). Given that CpxR_D51A_::His_6_ exhibited altered mobility in the Manganese(II)-Phos-tag™ acrylamide gel, we also confirmed the absence of phosphorylation using conventional SDS-12%-PAGE followed by visualization with the independent Pro-Q® Diamond phosphoprotein stain (Figure 2B). As expected therefore, Asp_51_ appears to be a genuine site of CpxR phosphorylation.

It is possible that substitution at Asp_51_ may cause an extreme allosteric effect that prevents CpxR phosphorylation at an alternative site. As phosphorylation on Asp residues is considered to be unstable, our initial approach had the goal to reduce phosphorylated Asp_51_ of CpxR∼P using sodium borohydride to a stable homoserine [33], [34] and to confirm this conversion by mass spectrometry. However, the yield of this reaction was very low so the approach was not continued. Instead, *in vitro* phosphorylation of CpxR was confirmed by intact mass determinations using ESI-MS (Figure S1). These experiments showed that the major product of *in vitro* phosphorylation of CpxR carried only one phospho group (Table 1). This is consistent with the model that Asp_51_ is the principal phosphorylation site of CpxR. In addition, the intact mass determination showed that CpxR∼P was sufficiently stable to be analyzed without converting Asp_51_ to homoserine by sodium borohydride reduction.

**Table 1:** Summary of mass determination of CpxR and CpxR∼P by ESI-MS.

Sample	Experimental mass	Theoretical mass
CpxR	27561±4	27560 (unmodified)
	27564±3	
	27556±4	
CpxR∼P	27640±3	27640 (singularly phosphorylated)
	27641±2	

### Phosphorylation-dependent binding of CpxR∼P to virulence gene promoters *in vitro*

**Figure 3:**
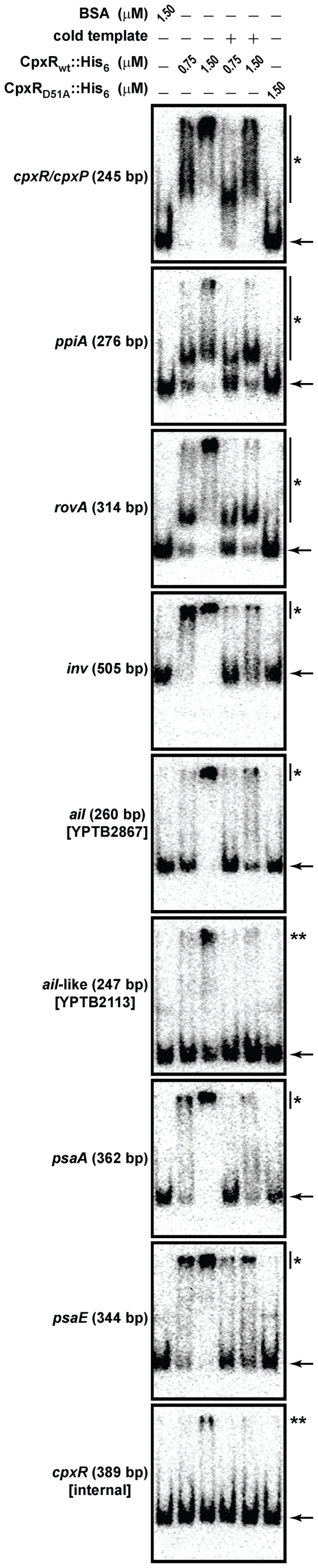
Phosphorylation-dependent binding of CpxR to DNA upstream of *Yersinia* virulence genes. Mobility shift assays were performed with purified CpxR_wt_::His_6_, and the non-phosphorylated mutant CpxR_D51A_::His_6_. Target DNAs were radiolabeled PCR fragments harboring the regulatory regions of *cpxR/cpxP*, *ppiA*, *rovA*, *inv*, *ail* (YPBT2867), *ail*-like (YPBT2113), *psaA* and *psaE*. An internal fragment of the *cpxR* gene was used as a negative control. The approximate size of each amplified PCR fragment is given in parentheses. Where indicated, the purified CpxR-His tagged variants were incubated with ‘hot’ DNA templates at the concentrations of 0.75 and 1.5 µM. All reactions were performed in the presence of the phosphodonor acetyl∼P and binding specificity was aided by the constant presence of BSA and non-specific single stranded DNA. EMSAs were further controlled by a reaction containing 1.5 µM BSA instead of CpxR or competition reactions in which excess ‘cold’ DNA template competed with ‘hot’ DNA for available CpxR∼P. The electrophoretic mobility of the ‘hot’ DNA fragments in the absence of bound protein is highlighted by an arrow, while DNA-CpxR∼P complexes are signified with an asterisk (*). In some cases, the extent of mobility shifted DNA was dependent on the CpxR∼P concentration. Two asterisks (**) indicate residual non-specific background binding noise and are not believed to represent bona fide binding targets of CpxR∼P.

Having established that CpxR∼P carries only one phospho group that is likely to be at Asp_51_, we now wanted to gain insight into the role of CpxR∼P in virulence gene regulation in *Yersinia*. In a previous study, we noted that the expression of genes encoding the potential adhesins Ail (*ail –* YPTB2867) and Ail-like (YPTB2113), as well as the confirmed adhesins pH6 antigen (*psaA*) and invasin (*inv*), are all influenced by loss of CpxA [20]. We made use of the purified CpxR_wt_ and CpxR_D51A_ His_6_-tagged variants derived from *Yersinia* CpxR and performed an EMSA to examine if CpxR∼P bound to radiolabeled PCR- amplified DNA control regions upstream of these *Yersinia* virulence genes. All regions of amplified target DNA included a minimum of ∼300 bp upstream and 40 bp downstream of the translational start codon. Our experiments were controlled by incorporating DNA encompassing the *cpxR/cpxP* divergent promoter and the *ppiA* promoter, both of which are known to bind CpxR∼P either from *Y. pseudotuberculosis* [20] or *E. coli* [12], [17], [42]. We also included the *rovA* gene encoding the transcriptional activator of invasin [22], [23], and the *psaE* gene, which encodes a regulator for pH6 antigen expression [43]. As anticipated from data obtained in our earlier study [20], *in vitro* phosphorylated CpxR_wt_ bound the promoters of *inv* and *rovA*, as well as those known CpxR∼P regulon members *ppiA* and *cpxR/cpxP* (Figure 3). Interestingly, at least two distinct migration shifts of *rovA*, *ppiA* and *cpxR/cpxP* template was observed when using different concentrations of CpxR∼P. This could suggest that these particular promoters contain multiple CpxR∼P binding sites of differential affinity so that several CpxR molecules bind cooperatively. This would be reminiscent of how CpxR∼P is thought to bind the promoter of *csgD* involved in curli biogenesis in *E. coli* [44]. Here, we also demonstrate for the first time a mobility shift of DNA specific to the promoter regions of *psaA* and *psaE*. Crucially, identical unlabeled (‘cold’) template DNA could compete for CpxR∼P binding. Although the degree of successful competition varied for each template, the addition of cold DNA did reduce the amount of radiolabeled (‘hot’) *cpxR/P*, *ppiA*, *rovA*, *inv*, *psaA* and *psaE* template DNA being retarded in migration during electrophoresis (Figure 3). Quite possibly the *ail* (YPTB2867) promoter also represents a target of CpxR∼P considering that the higher CpxR∼P concentration (1.5 µM) bound to all available free DNA resulting in the complete retardation of the hot template (the unbound signal at the bottom of the gel disappears). This is distinct from the reduced amount of *ail*-like (YPTB2113) promoter template shifted by equivalent amounts of CpxR∼P. In this case, significant signal representing free unbound *ail*-like hot template still exists at the bottom of the gel (Figure 3). In fact, this degree of ‘non-specific’ CpxR∼P binding was also observed in the negative control represented by an internal fragment of *cpxR* encompassing the nucleotides +32 through to +420 downstream of the translational start (Figure 3). Finally, binding by the non-phosphorylated CpxR_D51A_ variant was not observed in our assay conditions as evidenced by the absence of a migration shift for any hot DNA template (Figure 3). Taken together, this demonstrates that CpxR phosphorylation at position 51 is required for direct and efficient binding of CpxR∼P to the *rovA*, *inv*, *psaA* and *psaE* (and possibly also the *ail*) virulence gene promoters. On the other hand, modulation of expression of the Ail-like homologue (YPTB2113) by CpxR∼P is apparently not via direct binding, but must presumably involve at least one other unknown regulatory intermediate.

### Mapping the DNA binding site of CpxR∼P in the *rovA* promoter

**Figure 4:**
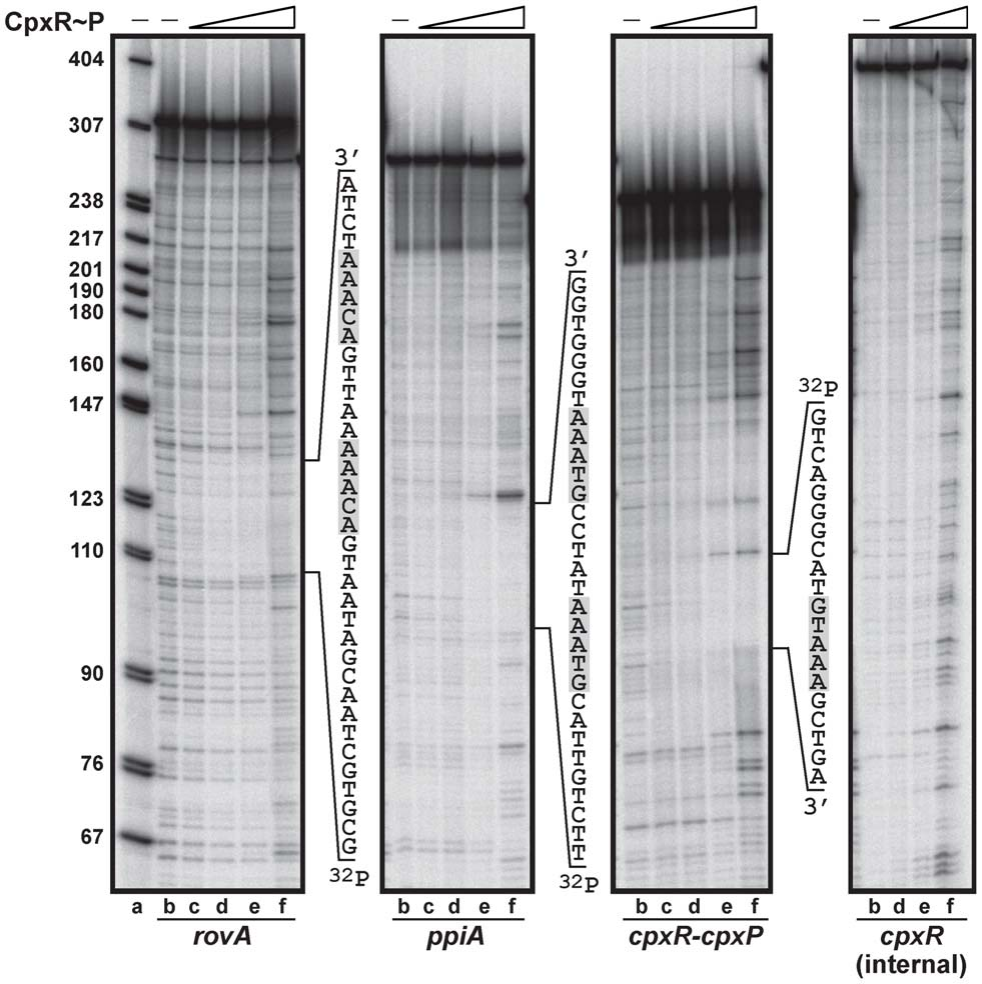
Mapping the CpxR∼P DNA binding site upstream of *rovA* by nuclease protection (footprinting) analysis. DNase I footprinting assays were performed to investigate the binding of CpxR∼P to a region within the *rovA*, *ppiA* and *cpxR-cpxP* promoters. These 314 base pair (bp), 276 bp and 245 bp fragments respectively, were labeled on the sense strand before being incubated with CpxR∼P at the following final concentrations: 50 nM, lane c; 100 nM, lane d; 200 nM, lane e; 400 nM, lane f. The absence of CpxR∼P in lanes a and b is indicated by ‘–’. Reactions were resolved by denaturing PAGE and analyzed with a Molecular Dynamics PhosphorImager. Labeled pBR322 DNA digested with MspI (New England Biolabs) was used as a size marker (lane a). An estimation of the protected sequence is given on the right hand side of the panels. Based upon the *E. coli* consensus sequence of 5′-GTAAA(N)_4–8_GTAAA-3, a putative CpxR∼P consensus binding site is highlighted in a gray box. A labeled internal fragment (389 bp) within the *cpxR* open reading frame served as a non-protected control.

RovA is a global regulator of *Yersinia* gene expression, being responsible for fine-tuning expression of a multitude of genes involved in general housekeeping, environmental survival and pathogenicity [24], [25], [26]. Levels of RovA need to be strictly controlled; dissecting these control mechanisms will therefore benefit our general understanding of how this pathogenic bacterium responds and adapts to its prevailing environment. In order to determine how CpxR∼P controls the levels of *rovA* transcription, we performed a nuclease protection (footprinting) analysis to map the DNA binding site of CpxR∼P in the *rovA* promoter. A protected sequence of the sense strand estimated to be 5′-gcgtgctaacgataatgacaaaaattgacaaatcta-3′ was identified within the P2 promoter region of *rovA* (Figure 4). We could also identify protected regions in the sense strand of *ppiA* promoter (5′-ttctgttacgtaaatatccgtaaatgggtgg-3′) and the divergent *cpxR-cpxP* promoter (5′-gtcagggcatgtaaagctga-3′) (Figure 4). As expected, a control sequence residing downstream of the *cpxR* start codon was not protected from DNase1 digestion. In *E. coli*, the consensus CpxR∼P DNA binding sequence is represented by 5′-GTAAA(N)_4–8_GTAAA-3′ [4], [42]. We manually inspected these DNase1 protected sequences for consensus CpxR∼P DNA binding motifs. A poorly conserved putative CpxR∼P binding site may lie in the protected region derived from the *rovA* promoter, while more obviously conserved consensus CpxR∼P binding motifs were observed in the protected sequence upstream of *ppiA* and between *cpxP*/*cpxR* (Figure 4).

**Figure 5:**
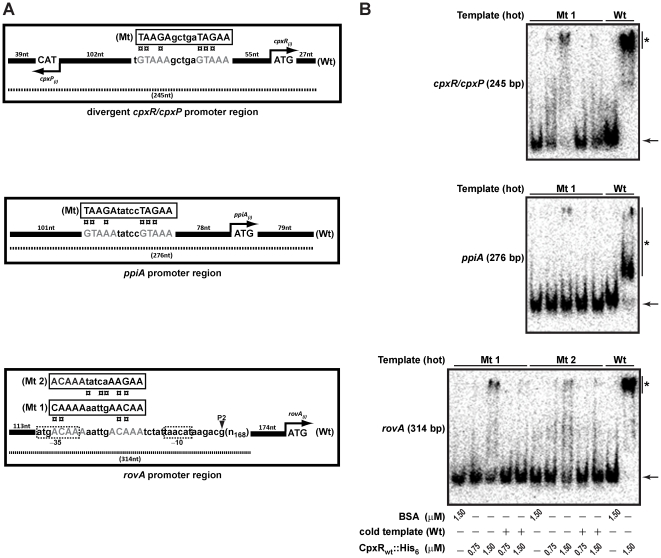
Site-directed mutagenesis of the CpxR∼P DNA binding site upstream of *rovA*. (A) As identified by DNase 1 foot-printing (see Figure 4), the potential CpxR∼P binding site sequence and position is shown relative to each ATG start codon within the regulatory regions of *rovA* and *ppiA* and the divergent *cpxR/cpxP* promoter (designated as ‘Wt’). Site-directed mutagenesis was performed to ‘shuffle’ the nucleotide sequence of each potential CpxR∼P binding site (designated as ‘Mt’). Since the initial mutation in the *rovA* sequence (‘Mt 1’) would potentially disrupt RNA polymerase binding because of an altered −35 region within promoter P2, a second mutation (‘Mt 2’) was performed in which the −35 region was left untouched. (B) Mobility shift assays with purified CpxR_wt_::His_6_ as outlined in the legend to Figure 3 were performed on radiolabled amplified DNA from these mutated templates. Once again, the electrophoretic mobility of the ‘hot’ DNA fragments in the absence of bound protein is highlighted by an arrow, while DNA-CpxR∼P complexes are signified with a single asterisk (*).

Based on the analysis of these ‘footprints’, we used sited-directed mutagenesis to shuffle the order of the nucleotides predicted to compose the CpxR∼P binding sites upstream of *ppiA*, *cpxR* (and *cpxP*) and *rovA* (Figure 5A). Since the CpxR∼P binding site upstream of *rovA* might overlap with the −35 box of the P2 promoter [45], we generated two scramble mutants in this region. The first was designated Mt 1, which included alteration of the −35 box sequence. In contrast, the second mutant (Mt 2) left the −35 box sequence intact. Significantly, an EMSA revealed a clear reduction in the retardation of PCR amplified fragments of the mutagenized DNA by CpxR∼P (Figure 5B; designated ‘Mt’). Collectively, these data indicate that the sequence 5′-ACAAA(N)_5_ACAAA-3′ overlapping with the −35 box of the P2 promoter and roughly located 360 nucleotides upstream of the *rovA* ATG start codon contributes to CpxR∼P binding. In addition, the 5′-GTAAA(N)_5_GTAAA-3′ sequences situated ∼80 and ∼55 nucleotides upstream of *ppiA* and *cpxR* respectively are also bona fide CpxR∼P binding sites.

### *In vivo a*ccumulation of CpxR∼P in the *Yersinia* cytoplasm reduces levels of RovA

**Figure 6:**
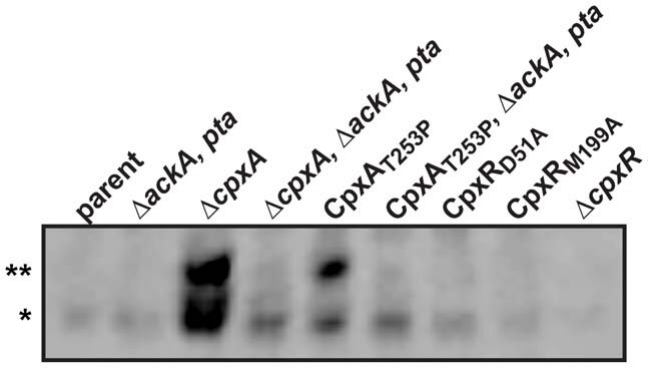
*In vivo* detection of phosphorylated and unphosphrylated CpxR. The Manganese(II)-Phos-tag™ acrylamide gel system was used to measure *in vivo* levels of CpxR∼P. Bacteria were cultured at 26°C in LB broth to stationary phase. After harvesting by centrifugation, bacteria were lysed with formic acid and samples rapidly fractionated on a Manganese(II)-Phos-tag™ 12.5% acrylamide gel and blotted with affinity purified anti-CpxR antiserum. Strains: parent, YPIII/pIB102; *ackA*, *pta* null mutant, YPIII69/pIB102; *cpxA* null mutant, YPIII07/pIB102; *cpxA*, *ackA*, *pta* null mutant, YPIII49/pIB102; cpxA101* encoding CpxA_T253P_, YPIII51/pIB102; cpxA101*, *ackA*, *pta* null mutant, YPIII74/pIB102; mutated *cpxR* encoding CpxR_D51A_, YPIII52/pIB102; mutated *cpxR* encoding CpxR_M199A_, YPIII46/pIB102; *cpxR* null mutant, YPIII08/pIB102. The single asterisk (*) reflects non-phosphorylated CpxR, while the double asterisk (**) indicates phosphorylated CpxR accumulated in the *Yersinia* cytoplasm.

We predict that binding of CpxR∼P to the *rovA* promoter restricts transcription. Therefore, elevating available CpxR∼P in the *Yersinia* cytoplasm in turn should reduce detectable RovA levels. To test this, we took advantage of work performed in *E. coli*, whereby phosphatase deficient CpxA_T253P_ encoded by the allelic variant designated cpxA101* generally lead to a constitutively active Cpx pathway [5], [14]. Although never directly tested, such a mutant should accumulate unusually high levels of CpxR∼P. To test this in *Yersinia*, we constructed an in cis cpxA101* mutation in *Y. pseudotuberculosis*. We then utilized the Manganese(II)-Phos-tag™ acrylamide gel system to measure *in vivo* levels of CpxR∼P. Formic acid-lysed bacteria grown in LB broth to early stationary phase (OD_600_ in the range of 0.85 to 0.95) at 26°C were fractionated on a Manganese(II)-Phos-tag™ 12.5% acrylamide gel and blotted with affinity purified anti-CpxR antiserum. Impressively high levels of both CpxR and CpxR∼P was detected in the cytoplasm of the *Yersinia* mutant completely lacking the *cpxA* allele (Figure 6). These two CpxR isoforms were also present in the cpxA101* background, although not to the same extent. Moreover, it seems that the vast majority of detectable CpxR is actually phosphorylated in this cpxA101* background. At present, reasons for why different levels and ratios of the two CpxR isoforms accumulate in these independent *cpxA* mutants are not clear. In contrast, CpxR was generally undetectable in parental bacteria or the *cpxR* mutants encoding the non-phosphorylated CpxR_D51A_ or the CpxR_M199A_ variant predicted to possess a defective winged helix-turn-helix domain (Figure 6). Given the noted toxicity of elevated CpxR∼P levels [4], [19], [20], accumulation of cytoplasmic CpxR∼P in the Δ*cpxA* and cpxA101* mutants does correlate with an inferior rate of growth (Figure S2A).

In the absence of CpxA phosphatase activity, CpxR∼P may generate via non-specific low molecular weight phosphodonors such as acetyl∼P [5], [15], [16], [17]. Acetyl∼P is derived from the phosphotransacetylase (Pta) – acetate kinase (AckA) pathway [46]. Therefore, to investigate if this phosphodonor contributes to accumulated CpxR∼P in the Δ*cpxA* null mutant and the gain-of-function cpxA101* mutant producing CpxA_T253P_, a full length Δ*ackA*, *pta* null mutation was introduced into these bacteria. Since mutants lacking both *ackA* and *pta* genes should be incapable of synthesizing acetyl∼P [47], this second-site mutation should therefore reverse the build-up of CpxR∼P exhibited by the Δ*cpxA* and cpxA101* mutants. Indeed, CpxR∼P no longer accumulated in the Δ*cpxA* and cpxA101* mutants also lacking the ability to synthesize acetyl∼P (Figure 6), which to some extent suppressed the inferior growth rate of the single Δ*cpxA* and cpxA101* mutants (Figure S2A). The singular Δ*ackA*, *pta* null mutant was also modestly growth restricted consistent with reports in *E. coli* (Figure S2A) [18], [48], [49]. However, it did not accumulate either non-phosphorylated or phosphorylated CpxR, being equivalent to parental bacteria where CpxR also remained below the level of detection in our assay (Figure 6). Hence, the Cpx pathway of *Yersinia* is subject to feedback inhibition that tightly restricts available CpxR∼P levels. However, in the absence of CpxA phosphatase activity, CpxR∼P readily accumulates by virtue of sensor kinase activity and/or small molecular weight phosphodonors associated with central metabolism.

**Figure 7:**
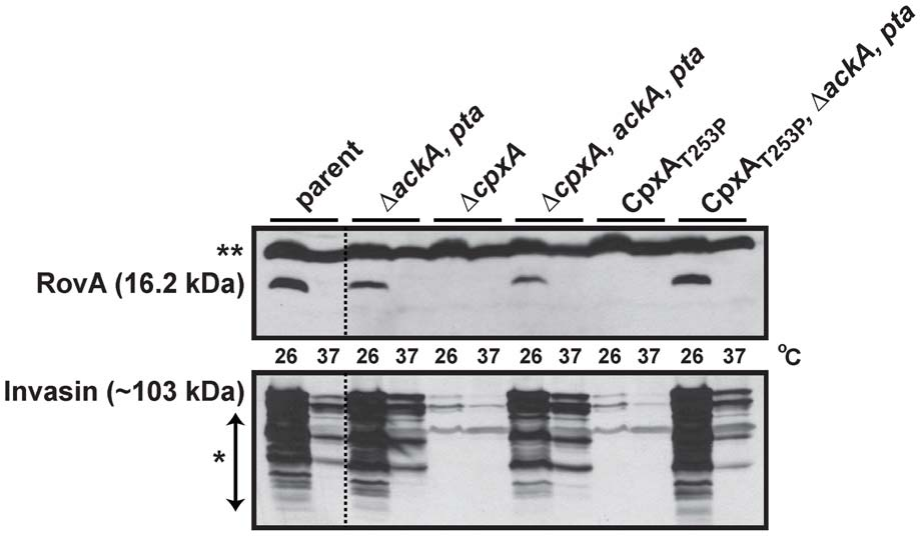
Cpx pathway activation influences the production of the RovA global regulator. RovA and invasin production was determined from protein isolated from early stationary phase bacteria grown in LB broth at either 26°C or 37°C. Protein was separated on a 12% SDS-PAGE and then identified by immunoblot analysis using polyclonal rabbit antiserum raised against RovA or invasin. Strains: parent, YPIII/pIB102; Δ*ackA*, *pta* null mutant, YPIII69/pIB102; Δ*cpxA* null mutant, YPIII07/pIB102; Δ*cpxA*, Δ*ackA*, *pta* null mutant, YPIII49/pIB102; cpxA101* encoding CpxA_T253P_, YPIII51/pIB102; cpxA101*, Δ*ackA*, *pta* null mutant, YPIII74/pIB102. Molecular weights shown in parentheses are deduced from primary sequence. The single asterisk (*) reflects typical invasin degradation products, while two asterisks (**) indicates a non-specific protein band recognized by the anti-RovA antisera that also serves as a convenient loading control.

Having established *Yersinia* mutants in which the *in vivo* levels of CpxR∼P were characterized, we used these to address whether elevated CpxR∼P levels restrict production of the RovA global regulator. RovA production from stationary phase bacteria grown in LB broth was analyzed by western blotting. Elevated levels of CpxR∼P accumulated in the Δ*cpxA* and cpxA101* mutants dramatically restricted RovA production (Figure 7). However, production to parental levels was restored in both mutants by the introduction of a second-site deletion of the *ackA* and *pta* genes (Figure 7). We also analyzed invasin, whose production is under RovA positive control. Not surprisingly therefore, invasin levels mirrored the pattern of RovA production in all strain backgrounds. Removal of the *ackA* and *pta* genes from parental *Yersinia* did not affect RovA or invasin production (Figure 7). We therefore conclude that cytoplasmic CpxR∼P accumulation is an important mediator of *Yersinia* virulence factor expression. The data also suggests that *Yersinia* low molecular weight metabolic intermediates, such as acetyl∼P, have the capacity to transfer high-energy phospho-groups to CpxR, and perhaps even other TCRS response regulators. Thus, this could be a way for *Yersinia* to effectively fine-tune global gene expression depending upon the prevailing environmental conditions such as nutrient supply.

### Elevated levels of the NlpE lipoprotein reduce invasin and RovA levels

**Figure 8:**
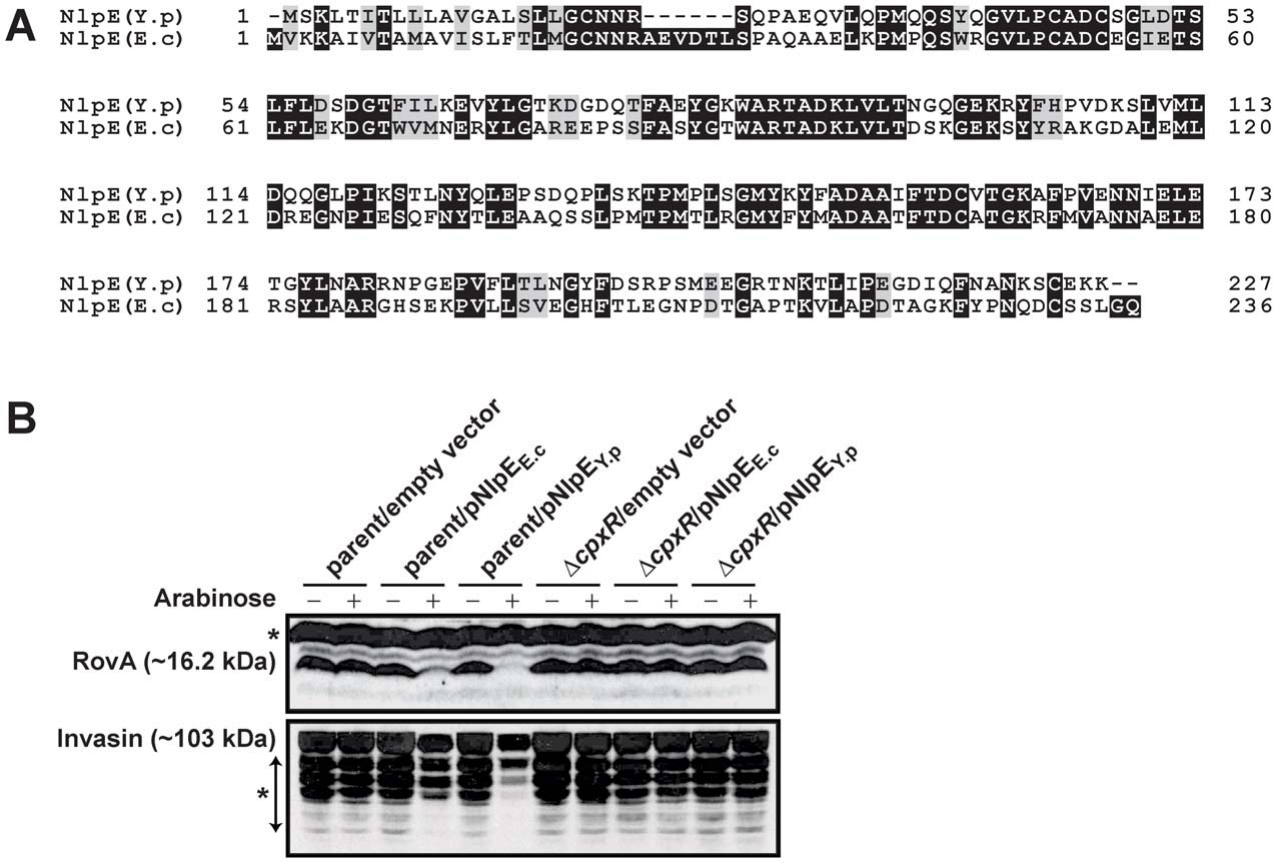
Inhibitory effect of NlpE overexpression. (A) A ClustalW2 alignment colored by Boxshade (ver. 3.21) between NlpE_Y.p_ (NCBI annotation YPK_1090) from *Y. pseudotuberculosis* YPIII and NlpE_E.c_ (b0192) from *E. coli* K-12 strain MG1655. (B) Sampling and detection of invasin and RovA at stationary phase in LB broth at 26°C followed the procedure detailed in the legend to Figure 7. Additionally, NlpE_E.c_ (derived from *E. coli*) or NlpE_Y.p_ (*Y. pseudotuberculosis*) over-production was controlled by the presence (+) or absence (−) of 0.2% (w/v) L-arabinose. The pBAD18 empty expression vector served as a control. The strains used were: parent with empty vector, YPIII/pIB102, pBAD18; parent with NlpE_E.c_, YPIII/pIB102, pND18; parent with NlpE_Y.p_, YPIII/pIB102, pJF027; *cpxR* null mutant with empty vector, YPIII08/pIB102, pBAD18; *cpxR* null mutant with NlpE_E.c_, YPIII08/pIB102, pND18; *cpxR* null mutant with NlpE_Y.p_, YPIII08/pIB102, pJF027. Molecular weights shown in parentheses are deduced from primary sequence.

Examination of the Cpx response in the absence of a functioning CpxA protein is artificial. We therefore wanted to examine the repressive effect of CpxR∼P on RovA production following activation of the Cpx response in which the signal transduction cascade is still intact. Simulating natural Cpx TCRS responsiveness has been achieved by the over-production of the *E. coli* NlpE (NlpE_E.c_) lipoprotein [50], [51]. We therefore transformed parent and CpxR defective *Yersinia* with pBAD18 derivatives encoding *E. coli nlpE* (termed pND18) or *Y. pseudotuberculosis nlpE* (NlpE_Y.p_ – pJF027) under the control of an arabinose inducible promoter. Since NlpE_Y.p_ shares less than 50% identity at the amino acid level with NlpE_E.c_ (Figure 8A), we considered that native NlpE could possibly be a better inducer of Cpx pathway activation in *Yersinia*. Bacteria were grown in the presence or absence of arabinose, and production of RovA and invasin was examined. Over-production of either NlpE_E.c_ or NlpE_Y.p_ in parental *Yersinia* caused a reduction in RovA and invasin production that was dependent on functional CpxR, because neither lipoprotein caused this decrease in the Δ*cpxR* null mutant (Figure 8B). Interestingly, NlpE_Y.p_ appeared to restrict RovA and invasin production to a greater degree. These data reinforce how Cpx pathway activation and the presence of CpxR∼P can restrict virulence factor production in *Y. pseudotuberculosis*.

### CpxR∼P DNA binding is required for RovA repression *in vivo*

**Figure 9:**
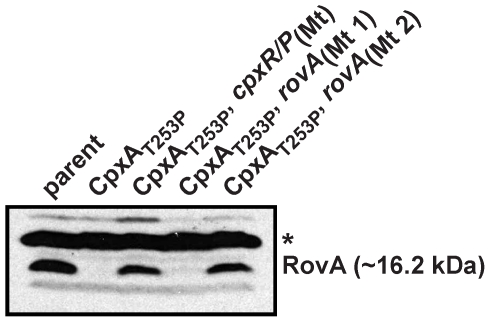
RovA produced from a mutant allele lacking the upstream CpxR∼P DNA binding site is no longer restricted by Cpx pathway activation. *Y. pseudotuberculosis* was grown until entry into early stationary phase at 26°C in LB broth. Bacterial cells were harvested and lysates prepared in loading buffer. Protein was separated on a 15% SDS-PAGE and then identified by immunoblot analysis using polyclonal rabbit antiserum raised against RovA. The molecular weight shown in parenthesis was deduced from primary sequence. The single asterisk (*) indicates a non-specific protein band recognized by the anti-RovA antisera. Strains: parent, YPIII/pIB102; cpxA101* encoding CpxA_T253P_, YPIII51/pIB102; *cpxR/cpxP*_(Mt)_ (shuffle mutation of the CpxR∼P binding site identified in Figure 4) placed in the cpxA101* background, YPIII115/pIB102; *rovA*_(Mt 1)_ placed in cpxA101*, YPIII120/pIB102; *rovA*_(Mt 2)_ placed in cpxA101*, YPIII121/pIB102.

We have identified a CpxR∼P binding site in the regulatory regions of three genes of *Yersinia*; *ppiA*, *cpxP/cpxR* and *rovA*. We have also shown the accumulated CpxR∼P levels result in restricted RovA production. Therefore, we wondered whether RovA repression by CpxR∼P could be relieved by modulating the CpxR∼P binding site within the *rovA* promoter. The same *rovA* ‘shuffle’ mutations (Mt 1 and Mt 2) used to identify CpxR∼P binding *in vitro* (see Figure 5) were introduced *in cis* into the chromosome of the cpxA101* mutant of *Y. pseudotuberculosis*. This background was chosen because the Cpx pathway is constitutively active, CpxR∼P accumulation is relatively high and the integrity of the *cpxRA* transcriptional unit remains intact. Bacteria were grown to early-stationary phase and the extent of RovA production was examined by immunoblot. RovA levels were essentially undetectable in the cpxA101* mutant and this mutant also harboring *rovA*_(Mt 1)_ (Figure 9). Significantly however, RovA levels were restored in the cpxA101*, *rovA*_(Mt 2)_ double mutant indicating that the ‘Mt 2’ mutation made the *rovA* promoter non responsive to Cpx pathway activation. Similarly, RovA levels were also restored in the cpxA101*, *cpxR/P*_(Mt)_ double mutant (Figure 9). In this strain, *cpxR* transcription is not induced limiting the accumulation of CpxR∼P (data not shown). To confirm these data, we performed semi-quantitative RT-PCR analysis to gain some insight into *rovA* transcription levels. Compared to the single cpxA101* mutant, *rovA* transcription was elevated in both cpxA101*, *rovA*_(Mt 2)_ and cpxA101*, *cpxR/P*_(Mt)_ double mutants (Figure S3). Interestingly, *rovA* transcription in the cpxA101*, *rovA*_(Mt 1)_ mutant was still low, which explains the absence of RovA detection by western blotting (Figure 9). This indicates that the ‘Mt 1’ nucleotide shuffling within the putative −35 region (see Figure 5A) predictably affected RNA polymerase promoter recognition and transcriptional output from the ‘P2’ *rovA* promoter [45]. Collectively, these data therefore identify nucleotides upstream of *rovA* that are critical for the *in vivo* recognition by CpxR∼P and the subsequent repression of *rovA* transcription. Despite our EMSA results indicating multiple CpxR∼P binding sites, we believe that this sequence in the ‘P2’ *rovA* promoter is primarily responsible for governing CpxR∼P-dependent *rovA* regulatory control.

## Discussion

Pathogenic bacteria experience diverse environmental conditions both inside and outside of a host. Multiple regulatory pathways are required to incorporate these diverse signals that then converge to fine tune spatial and temporal virulence gene expression. Herein, we report that the effect of CpxA-CpxR pathway activation in the enteropathogen *Y. pseudotuberculosis* is two-fold; not only does CpxR∼P serve to directly up-regulate the expression of genes encoding for periplasmic quality control factors, but it also down-regulates genes encoding virulence associated determinants – especially the global regulator RovA. In *Y. pseudotuberculosis*, the CpxA-CpxR TCRS therefore serves to coordinate the maintenance of bacterial envelope integrity with a reduction in virulence gene expression to ensure that vital energy resources are used to maximize the chances of survival in the prevailing environment.

How can CpxR∼P both induce and repress the expression of target genes in pathogenic *Yersinia*? A review of studies on other OmpR/PhoB family members provides some insight into the many assorted possibilities. CpxR∼P binding to target DNA may physically recruit the RNA polymerase. This principle is well-established for the phosphate starvation regulon, whereby PhoB∼P facilitates specific interactions between RNA polymerase and the *pho* promoters [52], [53]. CpxR∼P binding to target DNA may also alter DNA curvature that in turn modulates exposure of additional DNA binding sequences to provide access to secondary transcriptional activators or antagonistic repressors. This is no better illustrated than by the reciprocal interactions of OmpR∼P and integration host factor within the regulatory region of the *ompF* porin gene induced by changes in osmolarity [54], [55]. The orientation or the proximity of the CpxR∼P binding site relative to the transcriptional start may also influence activation or repression, especially if this influences CpxR∼P positioning with respect to the RNA polymerase or the binding sites for other activators or repressors. A strong correlation between genes under heavy CpxR-regulatory influence and the positioning of CpxR∼P binding sites within 100 base pairs upstream of the transcription start supports this notion [3]. Differences in relative binding affinities or the actual number of distinct binding sites within each promoter could also determine the degree of CpxR∼P influence. This has been used to explain the inverse regulation of the porin genes *ompC* and *ompF* by OmpR∼P [56]. It is also plausible that intrinsic promoter activity may influence CpxR∼P regulatory control. Indeed, such a mechanism may in part determine whether the RcaC response regulator activates or represses transcription of genes encoding products of cyanobacterial photosynthetic light harvesting antenna in response to ambient light color changes [57]. At this stage the mechanisms of CpxR∼P action are unclear, but future investigation will aim to reveal whether any of these complex scenarios dictate how CpxR∼P mediates both activation and repression of *Yersinia* gene expression.

Response regulator activation is thought to first require phosphorylation for the triggering of dimerization that is necessary for target DNA binding [58]. However, it is apparent that among the OmpR/PhoB subfamily, some members can bind DNA in the absence of initial phosphorylation. In fact, non-phosphorylated OmpR is proposed to first bind to DNA as a monomer [34], [59]. Phosphorylation then occurs to promote dimerization and facilitate DNA binding by the second monomer [59], [60]. The CovR response regulator of virulence and stress survival gene expression in *Streptococcus* spp. can also bind DNA as a non-phosphorylated monomer, although binding is enhanced by phosphorylation-dependent dimerization [61]. It is also notable that PhoP, a response regulator of *Salmonella enterica* that senses environmental Mg^2+^, dimerizes and binds DNA independent of phosphorylation [62]. In our own study, the non-phosphorylated CpxR_D51A_ variant was not able to bind DNA under the assay conditions used here. However, our previous results suggested the possibility of weak binding when nonphosphorylated CpxR was used in EMSAs with *rovA* and *inv* [20]. Thus, while phosphorylation significantly enhances CpxR binding to target DNA, we are unable to unequivocally conclude that binding cannot occur without it.

Mechanisms of *rovA* transcriptional regulation are complex (Figure S4). It chiefly involves a thermoregulated auto-amplification loop that overcomes H-NS/YmoA mediated silencing [22], [45], [63], [64]. However, an additional layer of positive regulation might incorporate LeuO, a LysR-like regulator [63], while negative regulation does involve another LysR-type protein, RovM [63], [65]. This negative regulatory loop probably senses nutritional status since its output is refined by participation of a carbon storage regulator system controlling *rovM* expression [66]. In addition, RovA activity is affected post-transcriptionally; temperature-dependent conformational changes render RovA less able to bind to target DNA sequences and to resist proteolysis by endogenous proteases [67]. Our data demonstrates another layer of RovA control; as a result of sensing ECS, accumulated CpxR∼P represses *rovA* transcription by a mechanism that involves direct binding to the *rovA* promoter. We will endeavor to pry apart this mechanism in order to understand its contribution to RovA control in relation to other regulatory players, especially those that converge on the 5′-UTR of *rovA* to fine-tune expression (Figure S5). Based on the CpxR∼P binding site overlapping with the −35 region of the ‘P2’ promoter, we currently speculate that CpxR∼P bound to the DNA may occlude appropriate positioning of the RNA polymerase holoenzyme and/or open promoter complex formation. Nevertheless, CpxR∼P has the ability to affect the expression of multiple genes [2], [3], [4], [68]. Thus, it also remains possible that CpxR∼P-dependent repression of *rovA* transcription involves additional indirect mechanisms; for example, by modulating *leuO* or *rovM* expression.

*Y. pseudotuberculosis* requires close eukaryotic cell contact in order to establish a host infection. Crucial to this are various adhesins located at the bacterial surface. CpxR∼P accumulation affects the expression levels of genes encoding several of these adhesins. While, CpxR∼P binds directly to the *inv* (invasin) promoter, the cornerstone of invasin control is positive regulation by RovA. We currently have no explanation for why CpxR∼P-dependent invasin regulation requires two mechanisms – one direct via binding to the *inv* promoter and the second indirect through binding to the *rovA* promoter. These two mechanisms may be a way to unite multiple overlapping signals to establish exquisite spatial and temporal control of *inv* expression – an important *Yersinia* virulence determinant. With respect to the control of other *Yersinia* surface adhesins, our DNA binding data suggests CpxR∼P might repress *ail* expression directly. Additionally, the controlled production of the pH 6 antigen appears to involve a complex mechanism requiring direct binding of CpxR∼P to the promoter of the tip adhesin gene *psaA* as well as to the promoter of *psaE*, encoding a membrane-associated protein required for transcriptional activation of *psaA* [43]. How PsaE mediates regulation of *psaA* transcription is currently unknown. Intriguingly, CpxR activation must also influence pH 6 antigen levels through an effect on RovA, since RovA binds to both *psaA* and *psaE* promoter regions [24].

In several respects the cpxA101*-encoding CpxA_T253P_ variant duplicates the effects of completely removing *cpxA*. In this context, it is curious that the cpxA101* and Δ*cpxA* full length mutants lacking *ackA* and *pta* both similarly failed to accumulate detectable CpxR∼P (see Figure 6). Unlike a complete *cpxA* deletion, one could have expected the gain-of-function cpxA* mutant to accumulate CpxR∼P, even in the absence of the Pta-AckA pathway, because it retains a genetically intact kinase component that can still phosphorylate CpxR [5], [11]. Significantly however, T253P substitution is known to hamper endogenous CpxA autokinase and CpxR phosphotransfer activity [5]. Thus, disruption of the Pta-AckA pathway in this background severely restricts an alternative means of CpxR∼P accumulation. In this context, it is worth questioning whether acetyl∼P is actually a bona fide *in vivo* phosphodonor responsible for kinase-independent activation of CpxR in *Yersinia*. While our data points in this direction, it was recently discovered that an intact Pta-AckA pathway, rather than acetyl∼P *per se*, may actually be more critical to CpxR activation [18]. Hence, removal of *pta* and *ackA* from *Yersinia* may conceivably erase a critical intracellular signal needed for CpxR activation. In *E. coli*, loss of the Pta-AckA pathway results in a plethora of diverse phenotypes and deficiencies ranging from defects in central metabolism, stress survival, proteolysis and chaperone function [46], [69]. Any one of these could potentially occur in *Yersinia* and thereby contribute to CpxR activation.

We made use of NlpE lipoprotein over-production, either from *E. coli* (NlpE*_E.c_*) or *Y. pseudotuberculosis* (NlpE*_Y.p_*) to stimulate an intact Cpx pathway. NlpE*_Y.p_* and NlpE*_E.c_* are about 49% identical at the amino acid level. Even though both proteins activated the Cpx pathway, NlpE*_Y.p_* did so to a greater extent. Exactly how the Cpx pathway is induced by NlpE is unknown, but it likely involves sensing of bacterial adhesion to surfaces [70]. Relay of this signal probably involves one or more structural changes in NlpE caused by external environmental stresses imposed during the adhesion process [71], [72]. Presumably this is mimicked in part by NlpE over-expression; an activating signal may result from erroneously assembled NlpE no longer transported to the outer membrane by the Lol system [71], [73]. Whatever the mechanism, it is striking for its molecular conservation and specificity. Of all the many lipoproteins produced by *E. coli*, only the over-expression of NlpE and one other – the inner membrane located YafY of unknown function – caused Cpx pathway activation [73]. Thus, comparative structure-function analyses of NlpE*_Y.p_* and NlpE*_E.c_* could potentially provide important insight into the activating mechanism of the Cpx pathway.

In an effort to provide experimental evidence for a phosphorylation of CpxR on Asp_51_, peptides were generated by fast enzymatic digestion. Commonly used proteases such as trypsin, chymotrypsin, and the endoproteinases AspN and GluC, were not suitable to generate peptides for a study of Asp_51_. All produced theoretically predicted Asp_51_-containing peptides with masses above 4000, which is relatively high for successful structural analyses by tandem mass spectrometry (MS/MS). Nevertheless, a protocol for fast digestion with trypsin and pepsin was developed. While MALDI-MS and ion trap MS combined with ESI and ETD ionization [74] both confirmed the identity of digested CpxR and CpxR∼P with high confidence, no peptides containing phosphorylated Asp_51_ or any other phosphorylation sites were detected (data not shown). This was even the case after enrichment of phosphopeptides using titanium dioxide [37], [38], [39]. Thus, we were unable to analyze phosphorylation of CpxR∼P by mass spectrometry. In part, this can be explained by the inability to generate specific peptides in the optimal mass range between 1200 and 2000 that would facilitate MS analysis.

In this study we have described how CpxR∼P accumulation has a direct negative effect on *rovA* expression in *Yersinia*. RovA is a global regulator targeting in excess of 60 genes in each of *Y. enterocolitica* and *Y. pestis* [24], [25]. As several of these genes may represent novel virulence determinants in these bacteria, the direct and indirect impact of Cpx pathway activation on *Yersinia* pathogenicity can be widespread. We see CpxR∼P as an ‘over-ride mechanism’ capable of a quick response to repress virulence gene expression in over-committed, fully-induced bacteria that suddenly find themselves in an unfavorable environment exposed to ECS where virulence factors are no longer useful for their survival – to continue to express them would waste precious energy resources.

## Supporting Information

Table S1 Bacterial strains and plasmids used in this study.
(RTF)

Table S2 Oligonucleotides used in this study.
(RTF)

Figure S1 Mass determination of intact CpxR and CpxR∼P by MALDI-MS. The respective CpxR and CpxR∼P peaks are indicated along with the experimental mass (Daltons).
(TIF)

Figure S2 Growth curves of various *Y. pseudotuberculosis* bacteria. Overnight cultures of parental and mutant bacteria were sub-cultured into fresh LB broth (time point 0 hours) and their growth during aerobic culturing with agitation at 26°C was monitored over a period of 9 hours by optical density measurement at 600 nm (A and B). Parent, YPIII/pIB102; YPIII07, Δ*cpxA*; YPIII08, Δ*cpxR*; YPIII46, mutated *cpxR* encoding CpxR_M199A_; YPIII49, Δ*cpxA*, Δ*ackA*, *pta* null mutant; YPIII51, cpxA101* encoding CpxA_T253P_; YPIII52, mutated *cpxR* encoding CpxR_D51A_; YPIII69, Δ*ackA*, *pta* null mutant; YPIII74, cpxA101*, *ackA*, *pta* null mutant; YPIII112, *cpxR/cpxP*_(Mt)_ placed in the parent background; YPIII113, *rovA*_(Mt 1)_ placed in the parent background; YPIII114, *rovA*_(Mt 2)_ placed in the parent background; YPIII115, *cpxR/cpxP*_(Mt)_ placed in the cpxA101* background; YPIII120, *rovA*_(Mt 1)_ placed in the cpxA101* background; YPIII121, *rovA*_(Mt 2)_ placed in the cpxA101* background. The asterisk (*) signifies modest to severe growth restriction in those bacteria with defects in the Pta-AckA biosynthetic pathway [18], [48], [49] (A) or in the CpxA sensor kinase that would be expected to accumulate toxic levels of CpxR∼P [4], [19], [20] (A and B). Note that despite being in a cpxA101* background, strain YPIII115/pIB102 grows quite well (B). Presumably, this is because CpxR∼P does not accumulate in this strain consistent with poor *cpxR* expression (data not shown) resulting from a shuffle mutation that makes the divergent promoter of *cpxR/cpxP* unable to bind CpxR∼P.
(TIF)

Figure S3 CpxR∼P DNA binding is required for repression of *rovA* transcription *in vivo*. For transcription analysis, crude semi-quantitative RT-PCR was performed on mRNA isolated from *Y. pseudotuberculosis*. Given the limitations of semi-quantitative RT-PCR, we attempted to increase the robustness of our assay by reverse transcribing mRNA isolated from bacteria grown at 26°C in LB broth to two different growth phases as measured by optical density at 600 nm; the first being an OD_600_ value between 0.45 to 0.55 (mid-logarithmic phase) and the second being an OD_600_ measurement between 0.85 to 0.95 (early-stationary phase). This was necessary to control for the altered growth rate of those bacteria expected to accumulate toxic levels of CpxR∼P (Figure S2B). Samples were subjected to RT-PCR with primers specific for *rovA* (plus RT). As an mRNA loading control, we analyzed the transcription of *rpoA* encoding for the α-subunit of RNA polymerase, which remained the same regardless of genetic background or phase of growth. To confirm the purity of the RNA isolated, PCRs with *rpoA* and *rovA* primer pairs was performed on template derived from RT reactions in which the enzyme was intentionally excluded (minus RT). PCR analysis on these samples indicated that the RNA isolation was essentially free of genomic DNA contamination. All images were acquired with a Fluor-S MultiImager (Bio-Rad) and analyzed with Quantity One software version 4.2.3 (Bio-Rad). DNA fragment sizes in base pairs are given in parentheses. Strains: parent, YPIII/pIB102; cpxA101* encoding CpxA_T253P_, YPIII51/pIB102; *cpxR/cpxP*_(Mt)_ (shuffle mutation of the CpxR∼P binding site identified in Figure 4) placed in the cpxA101* background, YPIII115/pIB102; *rovA*_(Mt 1)_ placed in cpxA101*, YPIII120/pIB102; *rovA*_(Mt 2)_ placed in cpxA101*, YPIII121/pIB102.
(TIF)

Figure S4 Established and potential mechanisms of Cpx-dependent modulation of *rovA* and *inv* expression. The Cpx pathway is a sensor of extracytoplasmic stress (ECS) [1]. However, the role of CpxR∼P as a central regulator of *Y. pseudotuberculosis* pathogenicity is also becoming apparent (this study) [19], [20]. CpxR∼P levels in the bacterial cytoplasm are manipulated by cognate CpxA kinase and phosphatase activity. This is even further affected by the CpxA-independent phosphorylation of CpxR via second messenger phosphodonors; the levels of which are somehow influenced by an intact AckA/Pta pathway. In turn, CpxR∼P binds directly to the *rovA* and *inv* promoters repressing transcriptional output (red line). This influence may also be augmented indirectly through as yet unknown (indicated by a dashed line and a ‘?’ symbol) regulatory links to other positive (green) and negative (red) factors controlling *rovA* and/or *inv* expression [22], [23], [45], [63], [64], [65], [66], [75], [76], [77]. Recently, H-NS was described to be a part of the CpxR∼P regulon [2]. However, our *in vitro* electrophoretic mobility shift analysis did not reveal any CpxR∼P bound to the *hns* or *ymoA* promoters (Liu and Francis, unpublished). Not shown in this diagram is the influence of CpxR∼P on *lon* expression [78], but this connection is still relevant given how RovA is subject to proteolysis by the Lon protease [67]. Elevated CpxR∼P levels also diminishes efficient T3S and the production of other ‘non-invasin’ adhesins by a mechanism(s) that are not yet understood (red dotted line).
(TIF)

Figure S5 Regulator binding sites in the upstream flanking sequence of *rovA*. *Y. pseudotuberculosis rovA* transcription is initiated from two sites (P1 and P2) upstream of the translational start codon (TTG – green) [45]. DNA sequences flanking P1 and P2 serve as binding sites for an array of regulators including H-NS (purple), RovM (dark blue) and RovA (red) [45], [65]. We have now shown herein that CpxR∼P (brown) also binds to DNA sequences that incorporate the −35 region of the P2 promoter. Based upon the binding site in the −35 region however, CpxR∼P could prevent access to the P2 promoter by the RNA polymerase holoenzyme. It is not yet known if or how CpxR∼P binding influences the binding of the other *rovA* DNA-binding regulators.
(TIF)
