# Supplementary material for: Phosphorylated CpxR Restricts Production of the RovA Global Regulator in Yersinia pseudotuberculosis
Source: PLoS One. 2011 Aug 18;6(8):e23314. doi: 10.1371/journal.pone.0023314 (PMC3158067; doi:10.1371/journal.pone.0023314)
Supplement: Table S1 — Bacterial strains and plasmids used in this study. (RTF) [file pone.0023314.s001.rtf]

Supplementary Table S1. Bacterial strains and plasmids used in this study

Strain or plasmid 	Relevant genotype or phenotype	Source or reference	
E. coli strains	
DH5	F, recA1, endA1, hsdR17, supE44, thi-1, gyrA96, relA1	Vicky Shingler	
S17-1ëpir	recA, thi, pro, hsdR-M+, SmR, <RP4:2-Tc:Mu:Ku:Tn7>TpR	[1]	
BL21(DE3) plysS	F, dcm, lon, ompT, hsdS(rB–, mB–), gal, ë(DE3), [pLysS, CmR]	Promega	
Y. pseudotuberculosis strains	
YPIII/pIB102	yadA::Tn5, PhoP–, KmR (parent)	Hans Wolf-Watz	
YPIII08/pIB102	pIB102, cpxR in frame deletion of codons 11 to 193, KmR	[2]	
YPIII52/pIB102	pIB102, cpxR allele encoding for the substation of D51A, KmR	This study	
YPIII46/pIB102	pIB102, cpxR allele encoding for the substation of M199A, KmR	This study	
YPIII07/pIB102	pIB102, cpxA in frame deletion of codons 41 to 449, KmR	[2]	
YPIII51/pIB102	pIB102, cpxA allele encoding for the substitution of T253P (cpxA101*), KmR	This study	
YPIII69/pIB102	pIB102, ackA, pta in frame double deletion from codon 17 of ackA through to codon 680 of pta, KmR	This study	
YPIII49/pIB102	ackA, pta in frame double deletion introduced into YPIII07/pIB102, KmR	This study	
YPIII74/pIB102	ackA, pta in frame double deletion introduced into YPIII51/pIB102, KmR	This study	
YPIII115/pIB102	Shuffle mutation of the CpxR~P binding site in the divergent promoter region between cpxR and cpxP (designated cpxR/P(Mt)) and introduced into YPIII51/pIB102, KmR	This study	
YPIII120/pIB102	Shuffle mutation of the CpxR~P binding site in the rovA promoter region (designated rovA(Mt 1)) introduced into YPIII51/pIB102, KmR	This study	
YPIII121/pIB102	Shuffle mutation of the CpxR~P binding site in the rovA promoter region (designated rovA(Mt 2)) introduced into YPIII51/pIB102, KmR	This study	
Plasmids			
pCR®4-TOPO®	TA cloning vector, KmR, ApR	Invitrogen	
pDM4	Suicide plasmid carrying sacBR, CmR	Debra Milton	
pJF007	~470 bp XhoI/XbaI PCR fragment of cpxAT253P (cpxA101*) in pDM4, CmR	This study	
pJF006	~1030 bp XhoI/XbaI PCR fragment of cpxRD51A in pDM4, CmR	This study	
pJF010	~1030 bp XhoI/XbaI PCR fragment of cpxRM199A in pDM4, CmR	This study	
pJF008	~455 bp XhoI/XbaI PCR fragment of ∆ackA, pta in pDM4, CmR	This study	
pJF033	~565 bp XhoI/XbaI PCR fragment of the shuffle mutation in the cpxR promoter region in pDM4, CmR	This study	
pJF037	~730 bp XhoI/XbaI PCR fragment of the 'Mt 1' shuffle mutation in the rovA promoter region in pDM4, CmR	This study	
pJF038	~730 bp XhoI/XbaI PCR fragment of the 'Mt 2' shuffle mutation in the rovA promoter region in pDM4, CmR	This study	
pBAD18	Expression vector, ApR	[3]	
pND18	SalI/HindIII PCR fragment of nlpE from E. coli cloned under the control of the arabinose promoter in the vector pBAD18, ApR	[4]	
pJF027	~770 bp EcoRI/XbaI PCR fragment of nlpE from Y. pseudotuberculosis cloned under the control of the arabinose promoter in the vector pBAD18, ApR	This study	
pET22b(+)	Expression vector, ApR	Novagen	
pKEC017	~700bp bp NdeI/XhoI PCR fragment of cpxR in pET22b(+) that creates a His(6) C-terminal fusion, ApR	[5]	
pJF024	~700bp bp NdeI/XhoI PCR fragment of cpxR encoding the mutation D51A in pET22b(+) that creates a His(6) C-terminal fusion, ApR	This study	
pJF025	~700bp bp NdeI/XhoI PCR fragment of cpxR encoding the mutation M199A in pET22b(+) that creates a His(6) C-terminal fusion, ApR	This study	


References

1. Simon R, Priefer U, Pühler A (1983) A broad host range mobilisation system for in vivo genetic engineering: transposon mutagenesis in Gram negative bacteria. Biotechnology 1: 787-796.
2. Carlsson KE, Liu J, Edqvist PJ, Francis MS (2007) Extracytoplasmic-stress-responsive pathways modulate type III secretion in Yersinia pseudotuberculosis. Infect Immun 75: 3913-3924.
3. Guzman LM, Belin D, Carson MJ, Beckwith J (1995) Tight regulation, modulation, and high-level expression by vectors containing the arabinose PBAD promoter. J Bacteriol 177: 4121-4130.
4. Danese PN, Snyder WB, Cosma CL, Davis LJ, Silhavy TJ (1995) The Cpx two-component signal transduction pathway of Escherichia coli regulates transcription of the gene specifying the stress-inducible periplasmic protease, DegP. Genes Dev 9: 387-398.
5. Carlsson KE, Liu J, Edqvist PJ, Francis MS (2007) Influence of the Cpx extracytoplasmic-stress-responsive pathway on Yersinia sp.-eukaryotic cell contact. Infect Immun 75: 4386-4399.
