# Supplementary material for: Phosphorylated CpxR Restricts Production of the RovA Global Regulator in Yersinia pseudotuberculosis
Source: PLoS One. 2011 Aug 18;6(8):e23314. doi: 10.1371/journal.pone.0023314 (PMC3158067; doi:10.1371/journal.pone.0023314)
Supplement: Table S2 — Oligonucleotides used in this study. (RTF) [file pone.0023314.s002.rtf]

Supplementary Table S2. Oligonucleotides used in this studya

Specific geneb	Oligonucletide pairc	
Mutagenesis		
cpxA101* (T253P) (471 bp)	pcpxA101a, 5´-ACG CTC GAG CTG CTG CTC TTG ATT GCG-3´ and pcpxA101b1, 5´-CCG GGT AAG TGG CGG GCG TAA CTC ATG GG-3´
pcpxA101c1, 5´-CGC CCG CCA CTT ACC CGG CTG C-3´ and pcpxA101d, 5´-ACG TCT AGA TGC ATT CTC CAG CAC ATC-3´	
cpxR (D51A) (1030 bp)	pcpxRa1, 5´-ACG CTC GAG AAC GAA CAT TGA CGC CAT-3´ and pcpxR-D51Ab, 5´-CAT AAT AGC AAG CAA TAA CAA GTC G-3´
pcpxR-D51Ac, 5´-TTA TTG CTT GCT ATT ATG ATG CCG C-3´ and pcpxRd1, 5´-ACG TCT AGA ACA GTG AGT TGA CGC GA-3´	
cpxR (M199A) (1030 bp)	pcpxRa1 and pcpxR-M199Ab, 5´-GAT GTG CGC ATC AAT CGC GCG GTC-3´
pcpxR-M199Ac, 5´-GCG ATT GAT GCG CAC ATC TCG AAC TTA CG-3´ and pcpxRd1	
ackA, pta (453 bp)	packAa 5´-AGC ACT CGA GAT TTC TCT ATC ATC ATG C-3´ and packAb 5´-TTT CAG GGA GGA GCT ACC GCA G-3´
pptac1 5´-GGT AGC TCC TCC CTG AAA GGG ATG CGC AAG CCG GTG-3´ and pptad1 5´-ACG TCT AGA CGA GCC TAC ATG GAT G-3´	
cpxR/cpxP promoter region (566 bp)	M-cpxR+ a, 5´-CTG ACT CGA GGT CGC CTT TCT TGT TCA TC-3´ and M-cpxR+ b, 5´-CTT CTA TCA GCT CTT AAT GCC CTG ACC CCA GTT TTA C-3´
M-cpxR+ c, 5´-TAA GAG CTG ATA GAA GTC ATG GAA TAC CGG CAG-3´ and M-cpxR+ d, 5´-AGT GTC TAG AGG GCA GTC AGC ATA ATC ACC-3´	
rovA promoter region (Mt 1; 731 bp)	M-rovA-a, 5´-TGG ATC TAG ACG ACG CTA AGT GTC AAT AAC-3´ and M-rovA-b, 5´-GAT TGT TCA ATT TTT TGC ATT ATC GTT AGC ACG CC-3´
M-rovA-c, 5´-CAA AAA ATT GAA CAA TCT ATT AAC ATA AGA CGT TGA G-3´ and M-rovA-d, 5´-AGA GCT CGA GAA CTA ATC GTG CTA GAT CAG-3´	
rovA promoter region (Mt 2; 731 bp)	M-rovA-a and M-rovA-b2, 5´-TAG ATT CTT GAT ATT TTG TCA TTA TCG TTA GCA C-3´
M-rovA-c2, 5´-AAA ATA TCA AGA ATC TAT TAA CAT AAG ACG TTG-3´ and M-rovA-d	
RT-PCR		
rpoA (328 bp)	prpoAa, 5´-GTT CGA CGC ACG CCA AGG TGA-3´ and prpoAb, 5´-ACG TCC TGC GGC TTG ACG AT-3´	
rovA (224 bp)	provAfor, 5´-GGC GCG CAT TAA TTG ACC ATC-3´ and provArev, 5´-AAT TCT CTT CGC ACG ACG ATC-3´	
Protein expression		
cpxRWT::his6 (708 bp)	pcpxR-Nde(ET), 5´-CAT ATG CAT AAA ATC CTA TTA GTT GAT G-3´ and pcpxR-Xho(HisET), 5´-CTC GAG TGT TTC TGA TAC CAT CA-3´	
cpxRD51A::his6 (708 bp)	pcpxR-Nde(ET) and pcpxR-Xho(HisET)	
cpxRM199A::his6 (708 bp)	pcpxR-Nde(ET) and pcpxR-Xho(HisET)	
nlpE(Yp) in pBAD18 (813 bp)	pYnlpe-for, 5´-GAA TTC ATA TGA ACG TTG ATA CAT TGC-3´ and pYnlpe-rev, 5´-TCT AGA ACT TGA ACT GCC GTG CG-3´	
Mobility shift assay and DNase I footprinting	
cpxR (internal, 389 bp)	pcpxRfor, 5´-GTG AAC TGA CGT CGC TGT TGA-3´ and pcpxRrev, 5´-TTG CAG GCA ATC AAC TTC CAG-3´	
cpxR/cpxP ('Wt/Mt'; 245 bp)	pcpxRb, 5´-GTC ATC ATC AAC TAA TAG GA-3´ and pcpxPb, 5´-AAC GAA CAT TGA CGC CAT AAC-3´	
ppiA ('Wt/Mt'; 276 bp)	E-ppiA for, 5´-GTC TGG GAT GTG GTG ACT CA-3´ and E-ppiA rev, 5´-GCT CAC CGG CAG CTA AAG-3´	
rovA ('Wt/Mt'; 314 bp)	E-rovA for3, 5´-CAT CTG GCT ATG TCG AGG-3´ and E-rovA rev3, 5´-TAA CCT AAT ACG AGT ATC CTC-3´	
inv (505 bp)	pinvfor1, 5´-TCA TCA AGG CAA CCA TCA GGA-3´ and pinvrev1, 5´-AGA AAC TCA CTG ATT GGC TGG-3´	
ail (260 bp) (YPTB2867)	E-ail for, 5´-AAC TCG ACA TGC CTC CC-3´ and E-ail rev, 5´-AAC ATG CAA TTA AAG AAG AGA-3´	
ail-like (247 bp) (YPTB2113)	E-ail2113 for, 5´-TGA CGT TTA TTA TTC TTG CG-3´ and E-ail2113 rev, 5´-CAT CAC TGG CAG CAT TCG-3´	
psaA (362 bp)	E-psaA for, 5´-CAT CCA GAA GTG GGT TAT A-3´ and E-psaA rev, 5´-GAC AGT AGA AGC GTT TGC-3´	
psaE (344 bp)	E-psaE for, 5´-TCA GTT AAT TCT GAC TGG C-3´ and E-psaE rev, 5´-CAC CTC ATT CTT GGA TAA AG-3´	
a	Primers were synthesized by DNA Technology A/S, Aarhus, TAG Copenhagen A/S, Copenhagen, Denmark or Sigma-Aldrich Sweden AB, Stockholm, Sweden.
b	The number of base pairs (bp) in parentheses indicates the approximated size of the amplified PCR fragment.
c	The EcoRI, NdeI, XhoI, SphI and XbaI restriction endonuclease sites are shown in italics. 
