# Supplementary figures and images for: Phosphorylated CpxR Restricts Production of the RovA Global Regulator in Yersinia pseudotuberculosis
Source: PLoS One. 2011 Aug 18;6(8):e23314. doi: 10.1371/journal.pone.0023314 (PMC3158067; doi:10.1371/journal.pone.0023314)

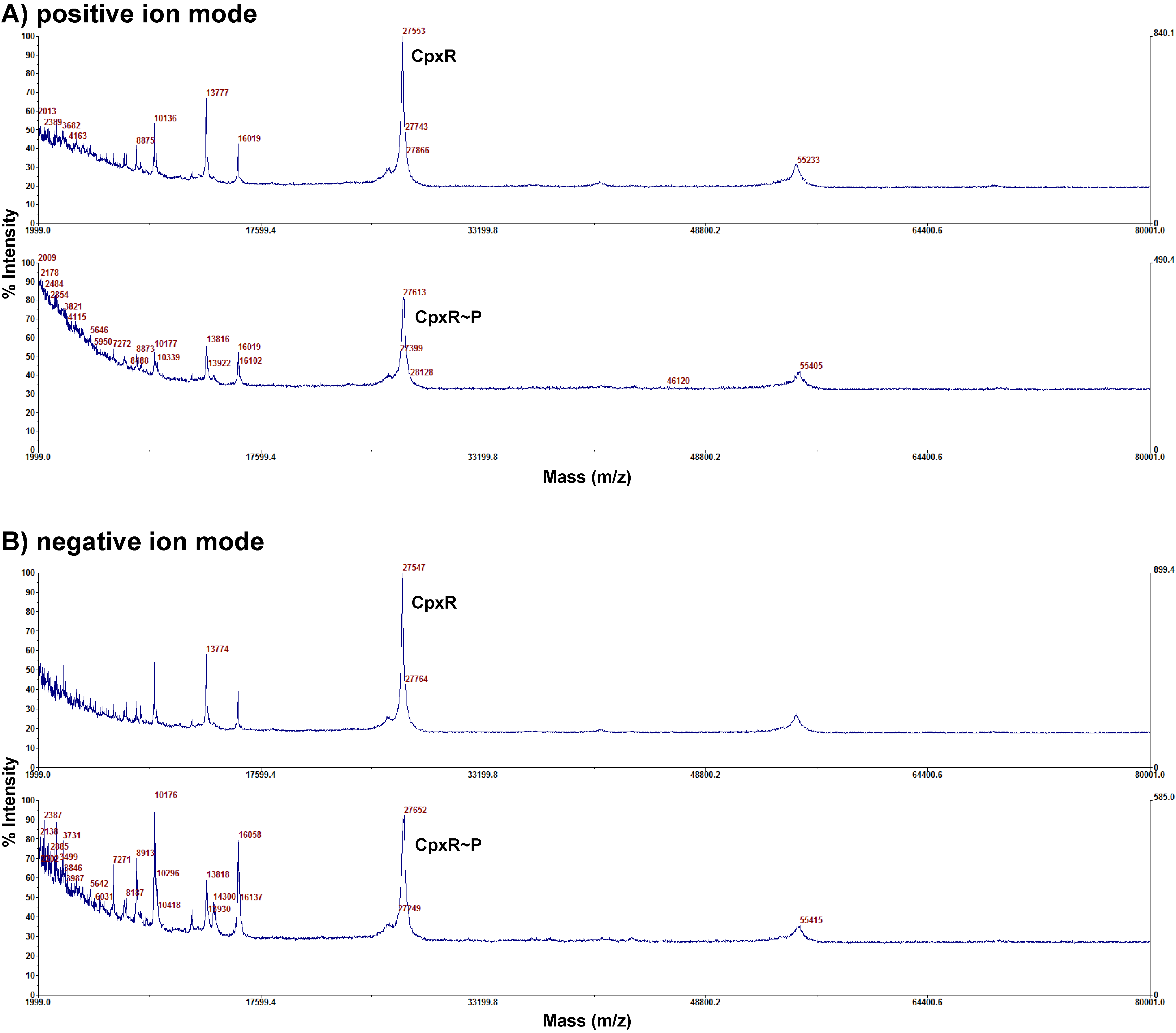

Supplement: Figure S1 — Mass determination of intact CpxR and CpxR∼P by MALDI-MS. The respective CpxR and CpxR∼P peaks are indicated along with the experimental mass (Daltons). (TIF) [file pone.0023314.s003.tif]

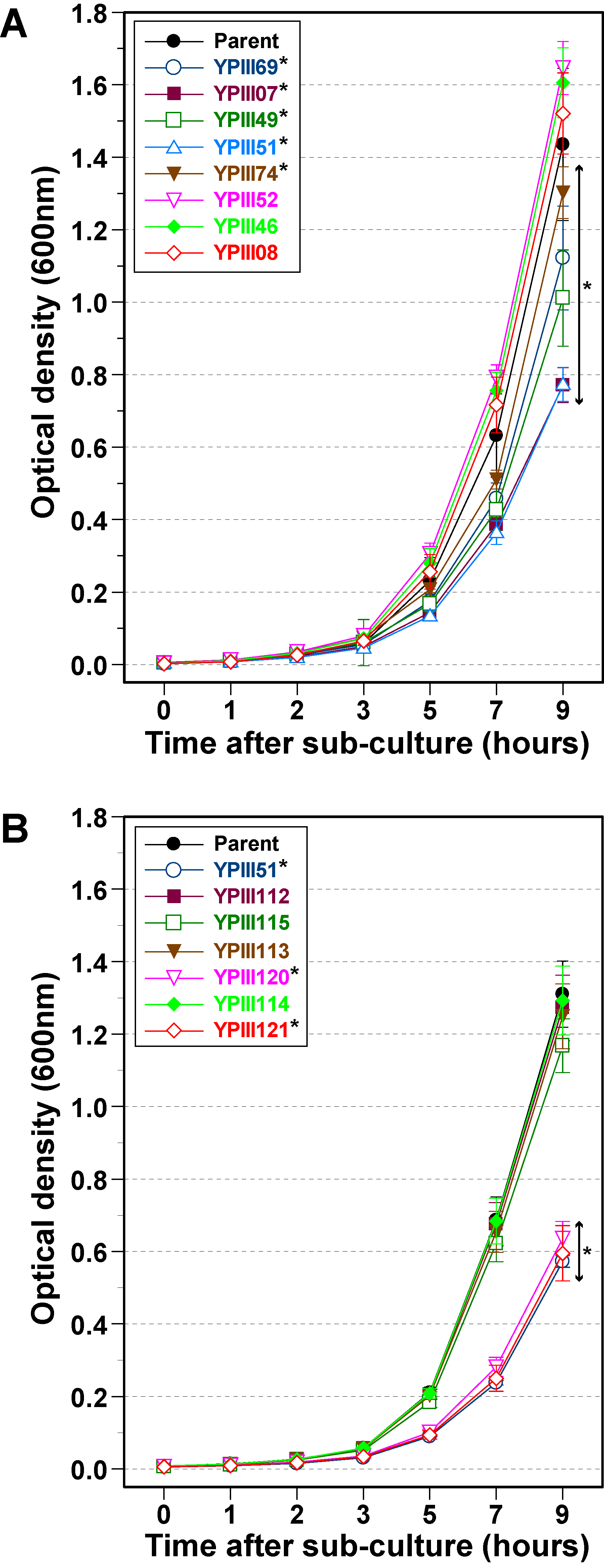

Supplement: Figure S2 — Growth curves of various Y. pseudotuberculosis bacteria. Overnight cultures of parental and mutant bacteria were sub-cultured into fresh LB broth (time point 0 hours) and their growth during aerobic culturing with agitation at 26°C was monitored over a period of 9 hours by optical density measurement at 600 nm (A and B). Parent, YPIII/pIB102; YPIII07, ΔcpxA; YPIII08, ΔcpxR; YPIII46, mutated cpxR encoding CpxRM199A; YPIII49, ΔcpxA, ΔackA, pta null mutant; YPIII51, cpxA101* encoding CpxAT253P; YPIII52, mutated cpxR encoding CpxRD51A; YPIII69, ΔackA, pta null mutant; YPIII74, cpxA101*, ackA, pta null mutant; YPIII112, cpxR/cpxP (Mt) placed in the parent background; YPIII113, rovA (Mt 1) placed in the parent background; YPIII114, rovA (Mt 2) placed in the parent background; YPIII115, cpxR/cpxP (Mt) placed in the cpxA101* background; YPIII120, rovA (Mt 1) placed in the cpxA101* background; YPIII121, rovA (Mt 2) placed in the cpxA101* background. The asterisk (*) signifies modest to severe growth restriction in those bacteria with defects in the Pta-AckA biosynthetic pathway [18], [48], [49] (A) or in the CpxA sensor kinase that would be expected to accumulate toxic levels of CpxR∼P [4], [19], [20] (A and B). Note that despite being in a cpxA101* background, strain YPIII115/pIB102 grows quite well (B). Presumably, this is because CpxR∼P does not accumulate in this strain consistent with poor cpxR expression (data not shown) resulting from a shuffle mutation that makes the divergent promoter of cpxR/cpxP unable to bind CpxR∼P. (TIF) [file pone.0023314.s004.tif]

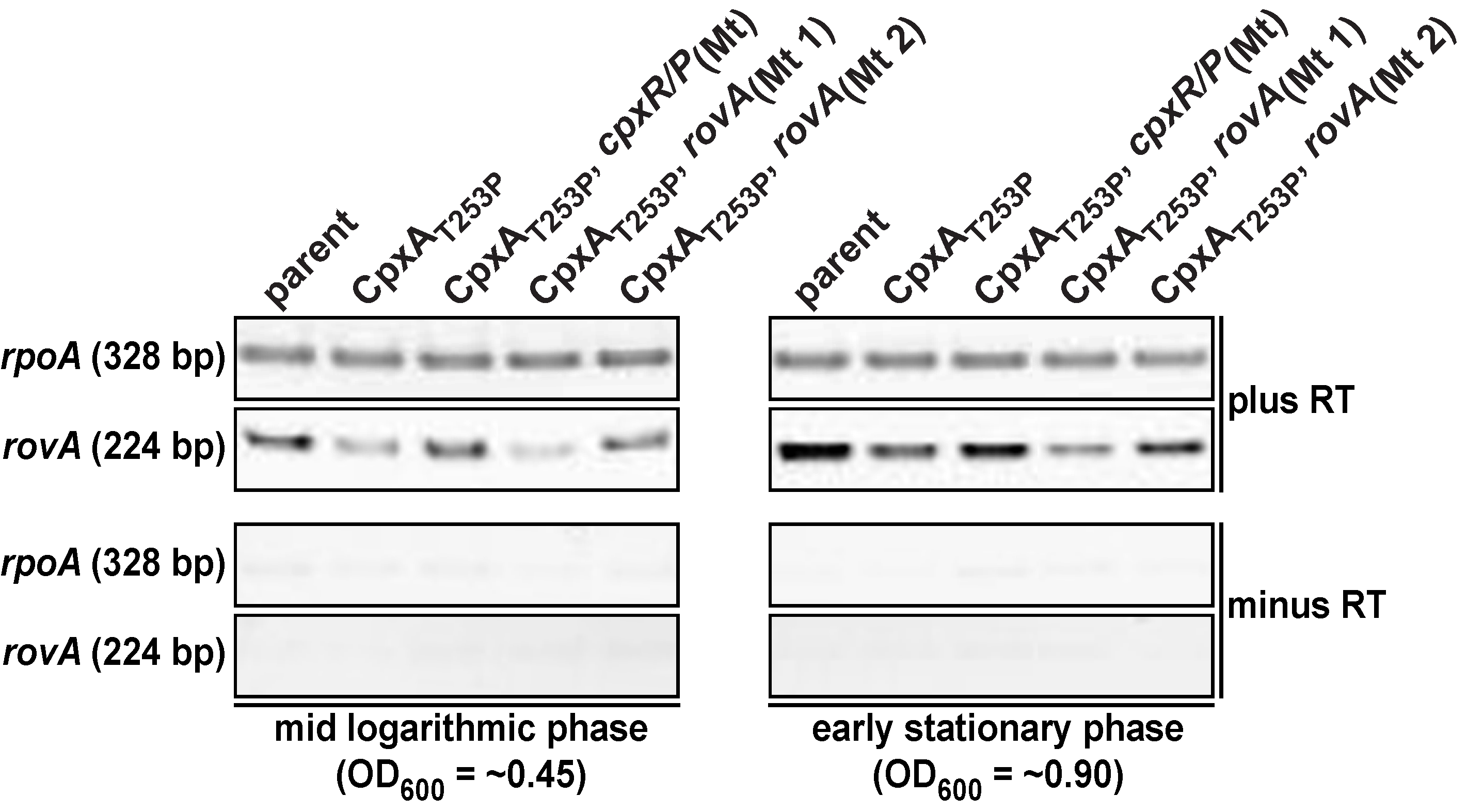

Supplement: Figure S3 — CpxR∼P DNA binding is required for repression of rovA transcription in vivo. For transcription analysis, crude semi-quantitative RT-PCR was performed on mRNA isolated from Y. pseudotuberculosis. Given the limitations of semi-quantitative RT-PCR, we attempted to increase the robustness of our assay by reverse transcribing mRNA isolated from bacteria grown at 26°C in LB broth to two different growth phases as measured by optical density at 600 nm; the first being an OD600 value between 0.45 to 0.55 (mid-logarithmic phase) and the second being an OD600 measurement between 0.85 to 0.95 (early-stationary phase). This was necessary to control for the altered growth rate of those bacteria expected to accumulate toxic levels of CpxR∼P (Figure S2B). Samples were subjected to RT-PCR with primers specific for rovA (plus RT). As an mRNA loading control, we analyzed the transcription of rpoA encoding for the α-subunit of RNA polymerase, which remained the same regardless of genetic background or phase of growth. To confirm the purity of the RNA isolated, PCRs with rpoA and rovA primer pairs was performed on template derived from RT reactions in which the enzyme was intentionally excluded (minus RT). PCR analysis on these samples indicated that the RNA isolation was essentially free of genomic DNA contamination. All images were acquired with a Fluor-S MultiImager (Bio-Rad) and analyzed with Quantity One software version 4.2.3 (Bio-Rad). DNA fragment sizes in base pairs are given in parentheses. Strains: parent, YPIII/pIB102; cpxA101* encoding CpxAT253P, YPIII51/pIB102; cpxR/cpxP (Mt) (shuffle mutation of the CpxR∼P binding site identified in Figure 4) placed in the cpxA101* background, YPIII115/pIB102; rovA (Mt 1) placed in cpxA101*, YPIII120/pIB102; rovA (Mt 2) placed in cpxA101*, YPIII121/pIB102. (TIF) [file pone.0023314.s005.tif]

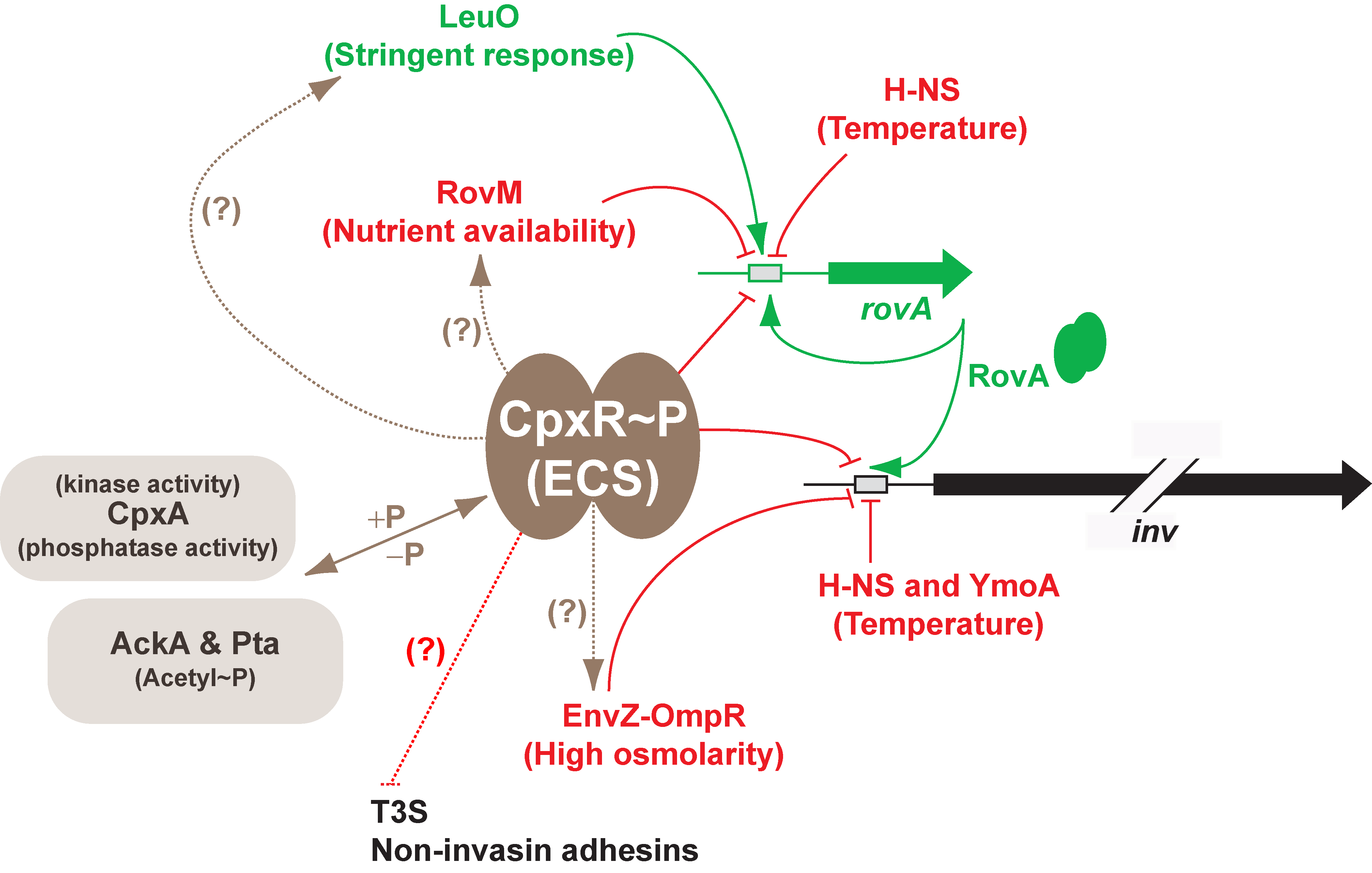

Supplement: Figure S4 — Established and potential mechanisms of Cpx-dependent modulation of rovA and inv expression. The Cpx pathway is a sensor of extracytoplasmic stress (ECS) [1]. However, the role of CpxR∼P as a central regulator of Y. pseudotuberculosis pathogenicity is also becoming apparent (this study) [19], [20]. CpxR∼P levels in the bacterial cytoplasm are manipulated by cognate CpxA kinase and phosphatase activity. This is even further affected by the CpxA-independent phosphorylation of CpxR via second messenger phosphodonors; the levels of which are somehow influenced by an intact AckA/Pta pathway. In turn, CpxR∼P binds directly to the rovA and inv promoters repressing transcriptional output (red line). This influence may also be augmented indirectly through as yet unknown (indicated by a dashed line and a ‘?’ symbol) regulatory links to other positive (green) and negative (red) factors controlling rovA and/or inv expression [22], [23], [45], [63], [64], [65], [66], [75], [76], [77]. Recently, H-NS was described to be a part of the CpxR∼P regulon [2]. However, our in vitro electrophoretic mobility shift analysis did not reveal any CpxR∼P bound to the hns or ymoA promoters (Liu and Francis, unpublished). Not shown in this diagram is the influence of CpxR∼P on lon expression [78], but this connection is still relevant given how RovA is subject to proteolysis by the Lon protease [67]. Elevated CpxR∼P levels also diminishes efficient T3S and the production of other ‘non-invasin’ adhesins by a mechanism(s) that are not yet understood (red dotted line). (TIF) [file pone.0023314.s006.tif]

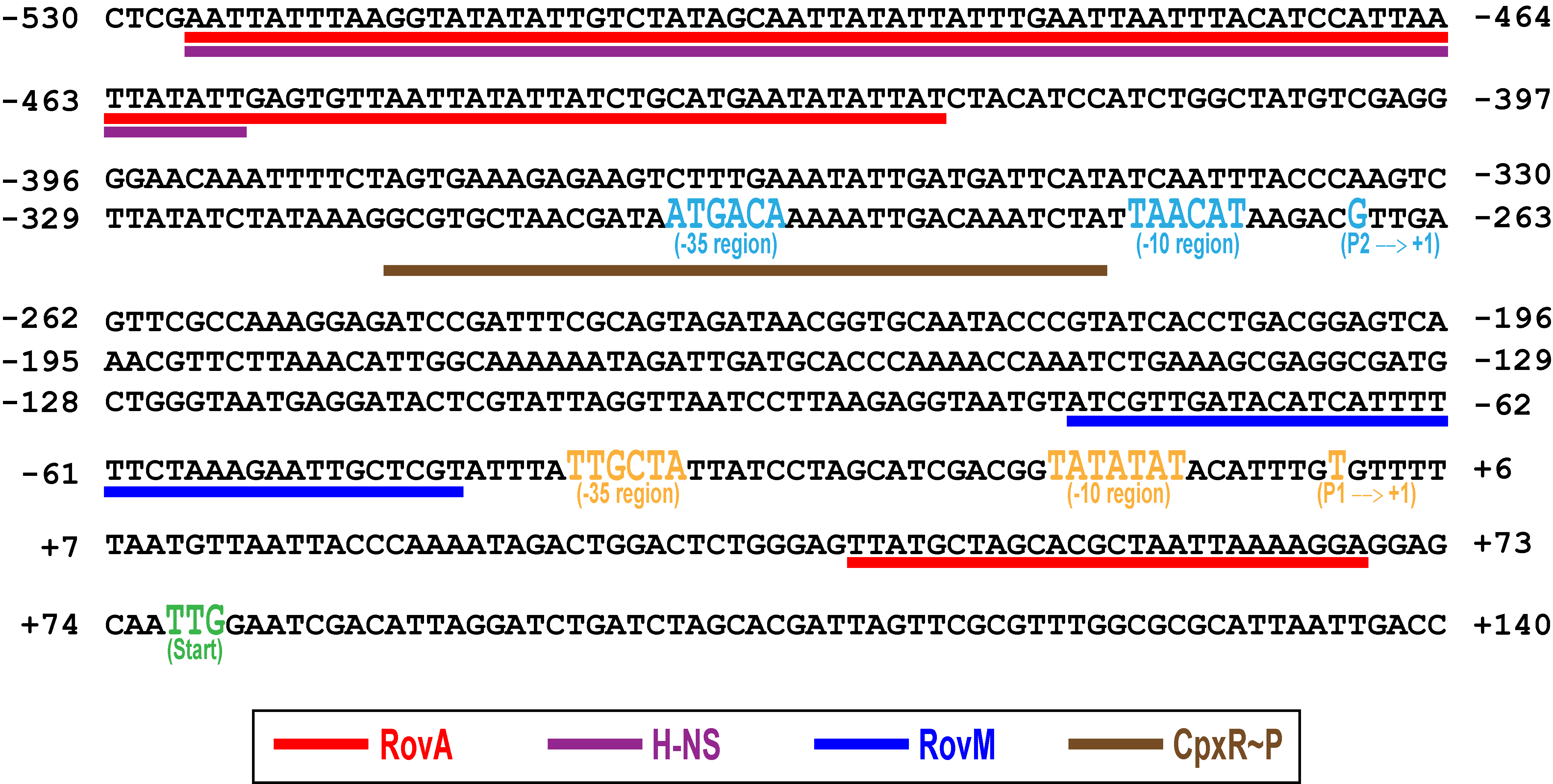

Supplement: Figure S5 — Regulator binding sites in the upstream flanking sequence of rovA. Y. pseudotuberculosis rovA transcription is initiated from two sites (P1 and P2) upstream of the translational start codon (TTG – green) [45]. DNA sequences flanking P1 and P2 serve as binding sites for an array of regulators including H-NS (purple), RovM (dark blue) and RovA (red) [45], [65]. We have now shown herein that CpxR∼P (brown) also binds to DNA sequences that incorporate the −35 region of the P2 promoter. Based upon the binding site in the −35 region however, CpxR∼P could prevent access to the P2 promoter by the RNA polymerase holoenzyme. It is not yet known if or how CpxR∼P binding influences the binding of the other rovA DNA-binding regulators. (TIF) [file pone.0023314.s007.tif]
